# Copper Corrosion and Biocorrosion Events in Premise Plumbing

**DOI:** 10.3390/ma10091036

**Published:** 2017-09-05

**Authors:** Ignacio T. Vargas, Diego A. Fischer, Marco A. Alsina, Juan P. Pavissich, Pablo A. Pastén, Gonzalo E. Pizarro

**Affiliations:** 1 Departamento de Ingeniería Hidráulica y Ambiental, Pontificia Universidad Católica de Chile, Santiago 7820436, Chile; itvargas@ing.puc.cl (I.T.V.); dafische.uc@gmail.com (D.A.F.); alsina@northwestern.edu (M.A.A.); ppasten@ing.puc.cl (P.A.P.); 2 CEDEUS, Centro de Desarrollo Urbano Sustentable, Santiago 7820436, Chile; 3 Facultad de Ingeniería y Ciencias, Universidad Adolfo Ibáñez, Santiago 7941169, Chile; juan.pavissich@uai.cl

**Keywords:** copper, corrosion, biocorrosion, drinking water, premise plumbing, MIC

## Abstract

Corrosion of copper pipes may release high amounts of copper into the water, exceeding the maximum concentration of copper for drinking water standards. Typically, the events with the highest release of copper into drinking water are related to the presence of biofilms. This article reviews this phenomenon, focusing on copper ingestion and its health impacts, the physicochemical mechanisms and the microbial involvement on copper release, the techniques used to describe and understand this phenomenon, and the hydrodynamic effects. A conceptual model is proposed and the mathematical models are reviewed.

## 1. Introduction

The lack of fresh and safe water will be one of the main problems worldwide in the next decades. Public health and the development of accurate analytic techniques have strengthened water quality standards, and new environmental issues have appeared. Indeed, ageing, deterioration of drinking water distribution systems and the associated growth of biofilms within the pipes have emerged as key infrastructure rehabilitation challenge [1].

For decades, copper has been the material of choice for piping used in household water distribution systems around the world. It is possible to classify the metallic materials for piping systems depending on their scale-forming characteristics. For example, copper and copper alloys are scale-forming materials unlike stainless steel [2]. Corrosion of copper pipes releases copper ions that may accumulate to concentrations high enough to create solid by-products which in turn affect the corrosion rate [2]. Copper pipes consist only of one chemical element while other scale forming materials (e.g., brass and bronze) are made of several elements. This variability in the composition, and consequently material characteristics alloys, does not facilitate the design of experiments and the understanding of leaching processes. Probably because of these reasons copper is the best studied material for domestic piping systems [2]. 

Even though copper is a noble metal, with wide application and experience of use in premise plumbing, it is affected by corrosion. The corrosion of copper pipes presents two fundamental problems: structural damage and human health risk from the release of copper-rich corrosion by-products (in dissolved or particulate form) into the drinking water. Reported cases of copper pipes failure, with its associated costs [3,4,5,6,7,8], along with population exposure to unsafe copper concentrations in drinking water [9,10,11] have motivated several scientific studies aimed to understand the mechanisms that trigger and control copper corrosion in premise plumbing [12,13,14]. 

Traditional studies of copper release in plumbing systems assume that the water extracted from a pipe follows a plug-type flow, and that the pipe surface does not interact with the bulk water under flow conditions. However, this approach underestimates the total mass of copper released from a pipe in a drinking water system [15]. Studies show that, under the presence of microorganisms, the hydrodynamic effects increase the release of copper [16,17]. This review presents a new conceptualization of copper release in drinking water systems, including time-dependence, biofilm and hydrodynamic effects.

## 2. Copper Issues

Copper pinholes in premise plumbing have been a problem for decades. The repair of the pinholes is usually a lengthy and expensive process. The detection of pinholes takes time because pipes are hidden, while the leaks may produce damage to structural elements and other valuable assets [18]. This problem has been reported all around the world. Large institutional buildings have suffered from leaks caused by biocorrosion in Scotland, South West England, Saudi Arabia, and USA [4]. During 2003, in Maryland, around 5200 cases of pinhole leaks were reported to the Washington Suburban Sanitary Commission (WSSC). After this, the WSSC decided to take measures by adding orthophosphate to the water and producing a consistent decline to about one leak report every month [19]. The addition of orthophosphate may also be used to maintain chlorine level in the water, thus preventing the growth of microorganisms [20].

One study, which reports the extension of the pinhole problem all over the USA [18], showed that during 1998 to 2004 each state had 1–20 reports of pinhole leaks. However, Maryland, Ohio, Florida, and California had a much larger number of pinhole leak reports. The study also suggests the main causes of pinholes in copper pipes in the USA are the high amount of chloride in water (30%).

According to the study by Farooqi, the estimated cost of a complete replumbing of copper pipes is around 2500 to 4000 US dollars for a two bath, two story house [18]. Repair costs may vary depending on the building. For example, in Scotland, around the late 1980s, the replacement of the defective pipes due to the extensive corrosion of hospital piping system was estimated to cost over £100 million [4]. The problem may be even bigger if the cause were due to bacteria in copper pipes, because of the sanitary problems it would mean to a hospital.

The pinholes are generated by the localized corrosion of the copper pipes. This means there is not a high amount of mass loss of the copper pipes, hence the release of copper ions into the drinking water is low. However, there are cases in which high amount of copper is released and pitting occurs simultaneously [12]. The extreme cases of high amount of copper release are known as the blue water phenomenon, since water turns blue. One of the highest copper concentrations reported reached 1 g/L [12].

Trace amounts of copper are essential for human diet; however, ingestion of copper could induce acute and chronic effects in some cases. While a high dose of copper induces acute effects in the gastrointestinal tract, chronic effects from long-term overexposure could result in liver damage because of copper accumulation [21,22,23].

Copper is ingested by humans mainly through food and drinking water. Drinking water can contribute to a significant proportion of the daily copper intake if it has flowed through copper pipes [24]. A provisional maximum tolerable daily intake (PMTDI) of 0.5 mg/d/kg of body weight was established by Joint FAO/WHO Expert Committee on Food Additives (JECFA) [18]. The WHO establishes a concentration of 2 mg/L of copper as a maximum value for drinking water. This value is based on acute and reversible gastrointestinal symptoms determined by epidemiologic studies conducted on controlled exposed populations [25]. In a study conducted in Shanghai (China), the average daily intake of Cu in drinking water was 21.12 µg/d which was only 0.01% of PMTDI for drinking water and food [11]. In another study conducted in Sweden, copper concentrations in first flush water from household taps were 0.17, 0.72, and 2.11 mg/L of Cu for the 10th, 50th, and 90th percentile, respectively. The estimated intake of copper for children who drank this water was 0.26–0.46 mg/d, which largely satisfied their daily copper requirement [26].

High, acute copper exposure induces toxic effects, consisting of acute symptoms (nausea, cramping, and vomiting) in the gastrointestinal tract [27]. The first and most frequent symptom reported is nausea, which is transient, appearing mainly within 15 min after ingestion [28,29,30,31]. The No Observed Effect Level (NOEL) was estimated in 2 mg/L of Cu, and the lowest observed adverse effect level (LOAEL) for nausea was determined to be 4 mg/L of Cu [29]. Diarrhea and abdominal cramps are rarely observed in concentrations below 12 mg/L of Cu [28,29,30,31]. Data from accidental or intentional ingestion of copper salts indicate that acute toxemia and possible death can occur when an adult ingests more than 4–400 mg of copper per kg of body weight [32].

The chronic effect of copper on human health has not been well studied, and it is uncommon on populations with no genetic defect in copper homeostasis [33]. A study conducted in reported no evidence of liver damage in a double-blind study of seven healthy individuals who consumed 10 mg/d of copper gluconate for a period of 12 weeks [34]. However, a case of chronic copper intoxication with no genetic disorder was reported on a young adult male, who consumed 30–60 mg/d of copper, which resulted in cirrhosis [35]. The toxicity by excess of copper is best demonstrated by Wilson’s disease, a genetic disease that is produced when the liver is unable to excrete copper and thus there is tissue accumulation, which can result in liver failure [36]. In addition, several neurological disorders such as Alzheimer’s, Parkinson’s, and prion diseases have been related to homeostatic alterations in brain copper levels [37,38,39].

Little research exists related to aesthetic problems of copper as a plumbing material. The distribution system has been reported as the major source of organoleptic problems [40,41,42]. Metallic taste was addressed as the second most important taste problem by water supplies [43]. Indeed, one of the major reasons for off-flavored water is metallic taste from copper or iron released from corroding pipes [10]. There is no agreement about the copper concentration that causes aesthetic effects; this is probably due to dissimilar sensitivities and competing effects on taste from other chemicals present in water. The U.S. EPA proposes a value of 1 (REVISAR) mg/L as a taste threshold concentration of copper [44]. On the other hand, the WHO proposes a value of 5 mg/L of Cu [25]. Table 1 presents the taste threshold concentration of copper found in three studies, using similar methodologies, but made under different conditions of population and water quality. These studies present important differences in the taste threshold concentration of copper for distilled or tap water. Cohen et al. [45] reported the highest threshold concentrations of 6.6 and 13 mg/L of Cu, whereas Dietrich et al. [10] presented the lower bounds of 0.5 and 0.2 mg/L of Cu for distilled and tap water, respectively. Thus, defining taste threshold concentrations for copper still requires more laboratory and real drinking water supplies research. 

## 3. Copper Processes in Drinking Water Systems

Copper release in drinking water pipes may be envisioned as the result of concurrent processes that may be classified into three groups: (1) electron transfer reactions; (2) copper speciation reactions; and (3) mass transfer processes. In the following paragraphs, we briefly review the mechanisms that control these processes. Finally, we discuss the role of a microbial biofilm, formed over the metallic surface, on the corrosion processes and the release of copper into the water. 

### 3.1. Electron Transfer Reactions

The oxidation of metallic copper is an electrochemical phenomenon of anodic and cathodic half reactions. There are several proposed mechanisms for the anodic reaction. Some studies propose a one electron mechanism on which copper reacts directly to form cuprite and then is further oxidized to Cu(II) species [47]. On the other hand, some authors propose that the Cu(II) species are formed directly [48]. The current understanding of these reactions suggest that the anodic half reactions produces mainly solid Cu(I) products, and soluble Cu(II) products, by three possible mechanisms, which may include intermediate steps [49]:

(1) Simultaneous mechanism
  2Cu + H_2_O → Cu_2_O + 2e^−^ + 2H^+^ (1)
  Cu → Cu^2+^ + 2e^−^ (2)
(2) Sequential mechanism
  2Cu + H_2_O → Cu_2_O + 2e^−^ + 2H^+^ (3)
  Cu_2_O + 2 H_2_O → Cu^2+^ + H_2_+2e^−^ + 2OH^−^ (4)
(3) Redeposition mechanism
  Cu → Cu^2+^ + 2e^−^ (5)
  Cu + Cu^2+^ + H_2_O → 2 Cu_2_O + 2H^+^ (6)

On the other hand, the cathodic half reaction is explained by the dissolved oxygen (DO) reduction [50]:

Dissolved oxygen reduction:

O_2_ + 4e^−^ + 4H_2_O → 4OH^−^ (7)

Copper has a strong tendency to react with DO. Based on thermodynamic calculations and kinetic studies, Ives and Rawson [50,51,52,53] propose that cuprite (Cu_2_O) is the first solid corrosion by-product formed on the pipe inner-surface. They proposed that the metallic surface is covered with a two-layer cuprite film. This conceptual model was called “duplex film model”. The first layer on the metal surface is compact and ~2 µm thick. The second layer of cuprite has high porosity. Due to these structural differences, the electrical resistance increases strongly at the interface between the compact and the porous cuprite films. This effect decreases the availability of electrons in the porous film, leading to further oxidation of the porous cuprite film and the subsequent formation of divalent copper scales [2]. 

The DO reduction semi-reaction is typically considered the main reduction reaction. However, it has to be taken into account that water chlorination adds stronger oxidants to this redox system, which produce a higher open circuit potential [54]. Therefore, the free chlorine reduction should be the dominant cathodic reaction in chlorinated waters. Cong and Scully studied this effect using cathodic polarization with DO or chlorine, demonstrating that chlorine has higher reduction rates than oxygen [55]. In this case, the reactions would be the following depending on the pH [56]:

HOCl + H^+^ + 2e^−^ → Cl^−^ + H_2_O (pH < 7.5) (8)

OCl^−^ + H_2_O + 2e^−^ → Cl^−^ + 2OH^−^ (pH > 7.5) (9)

Even though chlorine seems to be an important oxidizing agent to be considered in the conceptual model, its concentration and time of action in the piping system restrict its influence and significance compared with the DO present in aerated drinking water systems. However, the kinetics of the cathodic reaction when both free chlorine and DO are present have not been well studied and emerges as a key issue to determine the effect of both agents in the global process.

### 3.2. Copper Speciation Reactions

Precipitation-dissolution, complexation, and acid–base reactions govern copper speciation in the bulk water and in the metal–water interface. Scale formation reactions are dependent on pH, DO, temperature, and the amount and kind of ions present in the water. Generally, when the pH is higher than 6, cupric ions precipitate forming scales [2,57]. Pehkonen et al. [58] reported that the stability, thickness, and hence mass transfer properties of a corrosion by-product film are dependent on pH and DO.

X-ray diffraction and spectroscopy measurements have revealed the formation of Cu(I) and Cu(II) mineral phases, the former as cuprite (Cu_2_O) and the latter as either tenorite (CuO), cupric hydroxide (Cu(OH)_2_), malachite (Cu_2_(OH)_2_CO_3_), or other phases. The identity of the minerals formed at the solid–water interface strongly depends on the chemical composition (e.g., pH, HCO_3_^−^, SO_4_^2−^, PO_4_^3−^, and Cl^−^) and on physicochemical conditions (e.g., temperature and operational conditions) [2,15,57]. Temperature will be important for the formation of precipitates and metal solubility. In general, as the temperature increases, the amount of dissolved copper decreases, facilitating the formation of solids that precipitate on the pipe surface [59,60]. Although stagnation time in household systems varies according to local characteristics, the European and U.S. standards established a regulatory level within 6–12 h [61]. According to the German pipe rig standard (DIN 50931-1), which represents a standard consumption pattern of a kitchen tap in a four-person household, water stagnates 97% of the day and only flows 3% of the time. During stagnation conditions, metallic copper oxidation and scale precipitation-dissolution reactions may occur, but thermodynamic equilibrium is not expected before 12 h of stagnation [2].

Thermodynamics of copper corrosion supports the hypothesis that soluble corrosion by-product release from copper pipes is initially controlled by the solubility of cupric hydroxide [14,59]. The presence of anions in the water would catalyze the transition of cupric hydroxide to other less soluble phases that eventually passivate the corrosion process [12,62]. Using high resolution micro-X-ray diffraction, Merkel et al. [63] analyzed different pipe sections exposed for up to two years to high alkalinity groundwater (5.5 mM as HCO_3_^−^), adjusted to different pH. Cupric hydroxide was not found on the inner surface of the pipes, but the authors attempted to justify its absence by noting the instability of cupric hydroxide in the presence of anions such as carbonate. In neutral or slightly alkaline waters, cupric hydroxide has been reported as a metastable solid, ageing to less soluble phases such as tenorite or malachite [63]. In the presence of carbonate ions, malachite dominates the solid phase speciation of Cu(II) for pH between 5 and 9 [2].

During stagnation, copper concentration first increased to a maximum, then decreased, and finally leveled off at a constant concentration, which corresponds to the aqueous solubility of malachite [2]. This characteristic curve has been reported in previous works [2,63,64]. The curve is probably explained by the simultaneous action of electron transfer reactions (i.e., metallic copper oxidation increase the dissolved copper concentration above the solubility of malachite) and scale formation, which decreases the copper concentration until equilibrium is reached, expected after 30–48 h.

### 3.3. Mass Transfer Processes

Mass transfer processes control the flux of copper ions. Feng et al. [65] proposed that the limiting step of copper corrosion in drinking water is the diffusion of copper cations, probably Cu^+^, through the cuprite film. This effect has also been reported by other studies [66,67]. Mass transfer processes also control the copper flux from the surface of the pipe to the bulk water. Although most copper problems in drinking water piping have been related to soluble copper, the release of particulate corrosion by-products can also occur during a corrosion event. Indeed, flow conditions impose chemical and mechanical stresses at the metal–liquid interface, such as desorption of copper weakly bound to organic compounds contained in a biofilm matrix [3,68,69] and sloughing of micro- and nano-particles from corrosion by-products. The effect of the copper nanoparticles released due to hydrodynamic effects can increase copper release [70]. Nevertheless, a more recent study shows that higher Reynolds numbers do not change the total amount of copper released, but only the time scale of the process [16].

### 3.4. Microbial Involvement on Copper Mobility

The establishment of microorganisms in copper plumbing has to be considered an important factor influencing copper availability in drinking water. Several of metal mobilization and immobilization processes arise from microbial activity, including solubilization, chelation, precipitation, sorption, uptake, and intracellular accumulation. The extent of these mechanisms depends on the kind of microorganisms present and the water physicochemical conditions within water distribution systems. Drinking water typically undergoes a disinfection step along with a residual disinfectant concentration (e.g., chlorine-based compounds) along the distribution networks. However, this step is not efficient enough to control microbial growth. Conventional disinfection has been shown to reduce the concentration of planktonic bacteria, but to have a minor effect on bacteria growing attached to piping surfaces [71]. In copper pipes the growth of bacteria has been shown to be slower than in other materials [72], and it may be inhibited by copper toxic and bactericidal properties [73]. However, the prevalence of bacteria resistant to copper and disinfection [74,75] and a higher chlorine concentration declination rate in copper pipes [76] can lead to microbial persistence and accumulation.

Bacterial biofilms are usually found in drinking water pipelines, including copper plumbing [77,78]. A biofilm is a structured microbial community aggregated in a self-produced hydrated polymeric matrix and attached to a surface [79]. Biofilms are considered a prominent mode of microbial life. Under environmental stress this growth strategy increases tolerance and resistance to chemicals through a combined action of physicochemical and physiological phenomena [80]. The biofilm matrix is a complex network composed of the biofilm extracellular polymeric substances (EPS), including a variety of macromolecular components such as polysaccharides, proteins, glycoproteins, glycolipids, and extracellular DNA [81]. The EPS biopolymers interact between them and carry out functions giving ecological advantages to microbial communities thriving in challenging environments. The low content of nutrients in drinking water and the variable hydrodynamic conditions related with the transport of water within pipelines are harsh conditions for bacterial establishment. The biofilm structure confers mechanical stability to bacteria and the EPS has sorption properties that result in the accumulation of nutrients in distribution systems [78]. In copper pipes, biofilms constitute a protective barrier against copper; EPS components are capable of binding and sequestering copper ions limiting its mass transfer within the biofilm matrix [3,80]. The exopolymer production may vary depending on the bacterial community composition and the water chemistry [4,82], but in copper surfaces it has been shown to be enhanced with a high EPS to cell ratio [4]. The nature of the EPS functional groups is commonly anionic, determining a strong affinity for copper ions [3,4]. Moreover, some EPS are acidic and promote copper oxidation to ionic forms, which may also be captured in the biofilm [4]. Therefore, biofilms in copper plumbing form a reactive layer able to retain copper. 

Biocorrosion or microbially influenced corrosion (MIC) designates the accelerated deterioration of a material due to microbial presence and activity. In drinking water copper pipelines, microbial involvement in corrosion has been inferred from the extensive development of biofilms in corroding surfaces and demonstrated in laboratory scale experiments using bacterial pure cultures, biofilm extracts, and non-sterile water from corroding distribution systems [4,82,83,84,85]. MIC of copper pipes can highly increase cuprosolvency and enhance the release of particulated copper into the water [15,16,85]. Typically, MIC has been observed in soft water supplies with low levels of disinfection, causing pitting corrosion [4,6]. Pits in the presence of microorganisms are hemispherical. They are usually covered by a mound, or tubercle of malachite. Under this mound, there is a thin layer of cuprite, and possibly a layer of nantokite (CuCl). This thin layer has small openings and under this layer the biofilm can be found inside the pit [86,87].

**Figure 1:**
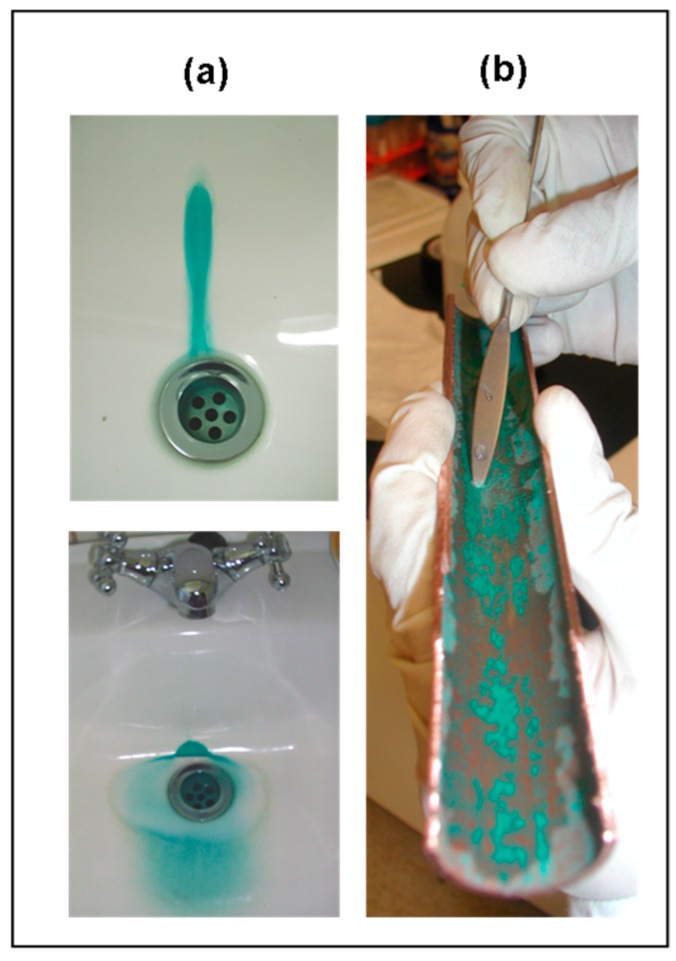
The effect of microbial biofilm formed on copper pipes household system: (**a**) bluish green stains in two sinks; and (**b**) two-year-old pipe affected by MIC.

Microorganisms have been also associated with the “blue water” phenomenon, in which particulate corrosion scales are released into the bulk water causing bluish green stains in domestic fixtures [88], and high copper concentrations (between 2 and 20 mg/L) [12,82]. Figure 1a presents bluish green stains formed in two sinks due to high copper concentration in the tap water. Figure 1b shows a two-year-old corroded pipe taken from a distribution system affected by MIC.

**Figure 2:**
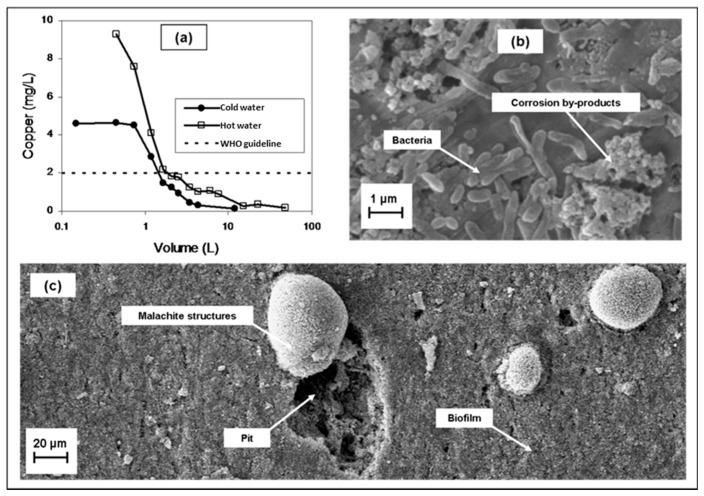
A copper pipe system affected by Microbiologically Influenced Corrosion (MIC): (**a**) copper release measurements; (**b**) Scanning Electron Microscopy (SEM) image of a biofilm formed on the pipe inner surface; and (**c**) SEM image of a pipe under MIC.

The role of biofilms in the corrosion of metals has been described based on several mechanisms occurring at the metal–liquid interface. The presence of a biofilm leads to the formation of microenvironments having different DO concentrations and oxidation-reduction conditions [89,90]. Within this heterogeneous environment, and under changing water chemistry and flow conditions, the transport by diffusion of chemical species from or towards the metal surface, the sorption and desorption of metals weakly bound to organic compounds, the structure and arrangement of the solid corrosion by-products, and the detachment of particles, are simultaneous phenomena involved in the overall corrosion process [90,91]. Figure 2a shows the effect on copper release of a biofilm formed on the metallic surface (Figure 2b,c). Copper concentrations over 9 mg/L (hot water, 40 °C) and 4 mg/L (cold water, 20 °C) were measured in the first liter of water sampled from a house affected by MIC.

A growing biofilm is a layer with variable diffusive and convective transport capacities, mainly given by the EPS spatial and chemical heterogeneity [4]. For instance, a mature biofilm matrix prevents the diffusion of DO to cathodic areas and of ions to anodic areas [91]. In copper plumbing, these microenvironments are suitable to be colonized by several copper-tolerant microorganisms, with a variety of metabolic features that contribute also to the progressive heterogeneity [4]. The development of microenvironments facilitates the metallic copper oxidation process, by imposing local conditions for the appearance of cathodic and anodic regions on the pipe surface. Copper soluble species released are sorbed by EPS components and corrosion by-products are accumulated through mineralization. This causes the formation of copper concentration cells and corrosion potentials [4].

The corrosion by-products are also affected by the biofilm matrix. The growth of a passivating layer above the metallic copper surface limits the transport of ions to and from the water, limiting the rate of the anodic half-reaction [65]. The dissolution of corrosion by-products changes in the inorganic chemical environment due to microbial activity, damages passivating layers and/or forces mineral replacement reactions that modify copper solubility [92]. The presence of bacteria also changes the morphology of corrosion by-products, for example malachite in the presence of biofilms form “hair like structures” instead of “spine like structures”, which could increase copper release under flow conditions [89].

Furthermore, operation of distribution systems imposes variable flux conditions (e.g., stagnation and flushing) that entail mechanical stress and the physical heterogeneity of the biofilm facilitating the detachment of protective corrosion by-products films. The sloughing of particles and desorption of weakly bound copper from the corrosion by-products and biofilm layers enhance the copper release into bulk water, suggesting that the reactivity of the biofilm matrix as a copper reservoir controls its release [15,16,89]. The pitting corrosion process under these circumstances is very difficult to control as corrosion cells are renewed periodically.

Even though most reports of biofilms on copper present them as detrimental to the pipes, they can also prevent corrosion. Garcia et al. showed that a mix of seven isolated bacteria can reduce the corrosion of copper pipes [93]. Another study showed that an isolated bacterium can reduce the corrosion produced by other bacterium [94], a recent mini-review explains these two roles bacteria can have with respect to corrosion [95]. Therefore, the study of the reactivity of the biofilm and the interactions of bacteria is a key factor for understanding MIC mechanisms and, as pointed out before, has multiple implications besides the sorption phenomena. Thus, to identify the role of this heterogeneous reactive matrix on copper mobility, it is important to use adequate techniques for water chemistry, surface, and microbial characterization.

## 4. Techniques for Water Chemistry and Surface Characterization

**Figure 3:**
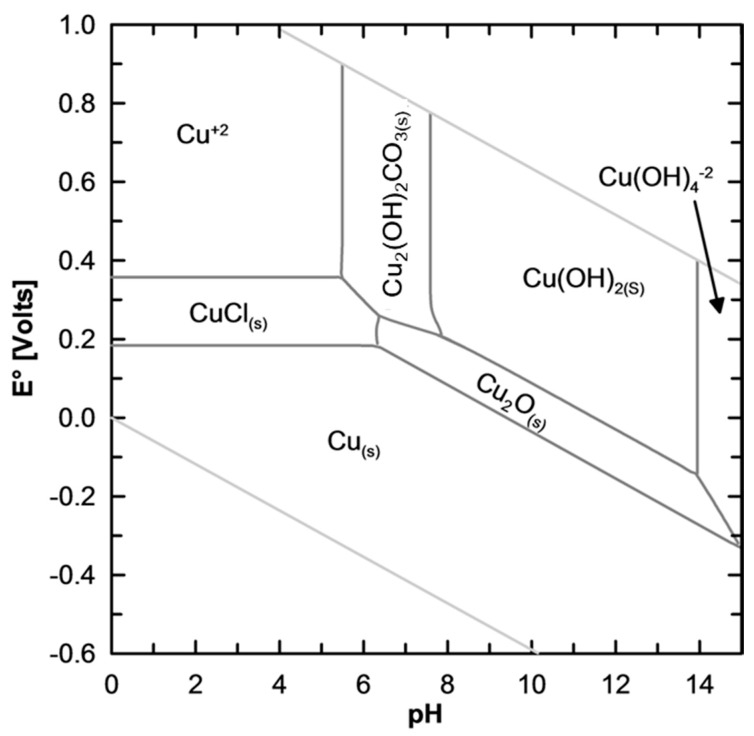
Pourbaix diagram of copper species for a closed system Cu-Cl-CO_3_. Calculations made considering Cu _TOTAL_ = 10^−5^ M, Cl _TOTAL_ = 8 × 10^−2^ M, CO_3 TOTAL_ = 10^−3^ M.

Characterization of water chemistry is a prerequisite to understand the mechanisms controlling distribution, mobility, and fate of aqueous and solid corrosion by-products [2,14,96]. Traditional characterization of water chemistry usually begins with the determination of parameters that give overall information about the system, and that are strongly dependent on local conditions. Such parameters include pipe diameter, temperature, electric conductivity, the activity of hydrogen ions in solution (measured as pH), DO, and the oxidation/reduction potential (ORP). Water constituents known to influence the nature of corrosion by-products include alkalinity, chloride, sulfate, phosphate, and organic matter [13,14,48]. As an example, consider the calculated predominance diagram for the Cu-Cl-CO_3_ system, presented in Figure 3. The diagram shows that a chloride concentration of 0.08 M favors the formation of nantokite at acid and slightly oxidizing conditions (pH < 6.3, and E° > 0.18 V vs. SHE), and this solid by-product has effectively been identified in corrosion pits formed under saline solutions [97,98]. On the other hand, malachite is shown to predominate at circumneutral pH and oxidizing conditions (E° > 0.3 V vs. SHE), an observation consistent with the predominance of this solid by-product in drinking water distribution systems [99,100]. It is important to notice that predominance diagrams consider thermodynamic equilibrium, so potentially metastable phases will not appear as predominant in the diagram, but can be actually present in the corrosion scale. Such is the case of atacamite, which is not shown in the predominance diagram, but has been identified in corrosion pits along with nantokite in saline solutions [97,98]. Conversely, other phases can be thermodynamically favored to appear in the predominance diagram, but have kinetic constrains that prevent them to appear until the weathering stages of the corrosion process. Such is the case of tenorite, which was discarded from the diagram owing to its poor relevance during the initial stages of the corrosion process [14,101].

Consequently, it is important to understand which techniques and sampling protocols are used to characterize water chemistry, corrosion by-products, and biological films formed on the pipe inner surface. The following paragraphs present different techniques and protocols for measuring particulate and soluble copper in water and for characterizing the inorganic and organic films that control the copper release into the water. 

### 4.1. Techniques and Protocols for Measuring Total and Soluble Copper in Water 

Atomic absorption spectroscopy (AA) and Inductively Coupled Plasma Atomic Emission Spectroscopy (ICP-AES) (ICP) had been widely used for measuring total and soluble copper in water in corrosion of copper pipes studies [14,15,48,57,102]. Total and dissolved copper has been also measured using the bicinchoninate method with a spectrophotometer [13,15,48,102]. This method has an analytical window of 0.04–5.00 mg/L. In addition, ion selective electrode of Cu^2+^ has been used to measure cupric ions in solution [103,104], being a key technique for identifying copper sorption/desorption on organic compounds [104]. The combination of these analytical techniques provides information about the speciation of copper in water, helping to elucidate the processes that occur at the metal–liquid interface. 

**Figure 4:**
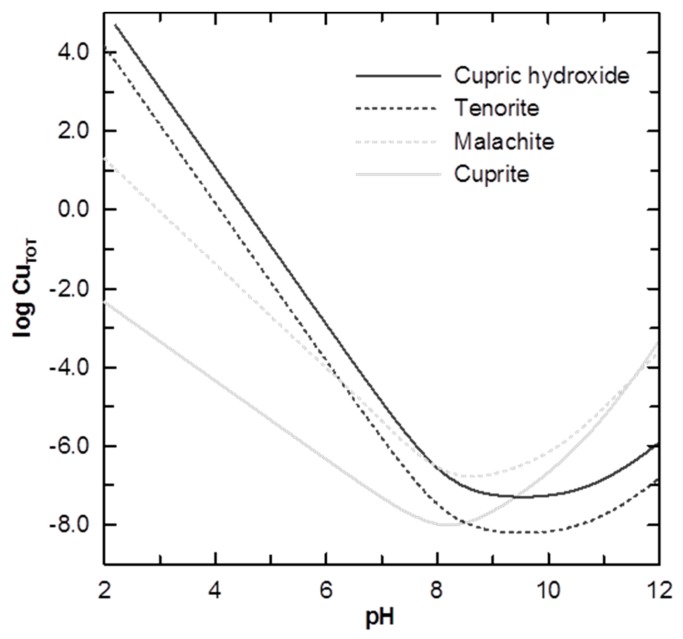
Example of hindered interpretation of copper solubility owing to copper nanoparticles measured as dissolved copper. Cupric hydroxide dominates solubility at pH 7. However, if copper nanoparticles are contributing with as low as ~0.01 mM (measured as dissolved copper), then control over copper solubility can shift from cupric hydroxide to either malachite or tenorite. Calculations made considering CO_3 TOTAL_ = 10^−5^ M, E° = 0.18 V.

During sampling, a common operational definition is the distinction between dissolved and colloidal constituents, given by the pore-size of the membrane used to filter the water sample. Typical pore sizes for membranes are 0.45 or 0.2 µm, however it is well documented that copper nanoparticles can reach diameters of around 50 nm [70,105]. Although we have found no evidence supporting formation of copper nanoparticles during corrosion events outside the laboratory, a cautious interpretation should assume that these particles may be occurring in the system, and their presence (larger than 0.01 mM at pH ~ 7) could lead to misinterpretation of the solid phase controlling the solubility of a given system (Figure 4). Additionally, toxicology tests of copper are usually performed on either dissolved or insoluble copper [106], so health effects owing to copper nanoparticles can be misinterpreted as the result of exposure to soluble copper.

### 4.2. Electrochemical Techniques

Electrochemical techniques are typically performed using a three-electrode electrochemical cell configuration which includes: a copper working electrode, a platinum auxiliary electrode, and a reference electrode such as silver/silver chloride (Ag/AgCl) [58,107,108]. Using electrochemical techniques, the instantaneous corrosion rate can be measured, instead of the cumulative corrosion rate. Feng et al. [65,109] conducted a study using synthetic tap water and copper coupons, and showed that the corrosion rate measured by mass loss and polarization are comparable. In this study the corrosion rate diminished as the copper was exposed to synthetic tap water from 2.1 µA/cm^2^ at 10 h of exposure, to 0.29 µA/cm^2^ after 45 h, and it stabilized from 120 to 500 h at 0.11 µA/cm^2^. Valcarce et al. [110] obtained similar results, where the corrosion rate decreased from 0.81 µA/cm^2^ after 2 h to 0.14 µA/cm^2^ after 192 h. In this study, the corrosion rates were measured using three different electrochemical techniques: polarization, polarization resistance, and Electrochemical Impedance Spectroscopy (EIS). The results for the techniques were similar except for the polarization at 2 h of exposure, which was about one half when compared to other techniques.

Electrochemical techniques have showed that the corrosion rate of copper depends also on several water quality parameters. Through potentiostatic polarization scans and Tafel slopes analysis Pehknonen et al. [58] found that the corrosion rates and the amount of copper released into the water depend on pH, DO, buffering capacity, ORP, and conductivity, as well as the concentrations of chloride and dissolved inorganic carbon. In this study, the corrosion rate decreased about one order of magnitude when the pH was increased from 5.5 to 8, and, from 8 to 9, the corrosion rat was stable. Combining corrosion current, potential measurements, and electron microscopy observations of copper coupons tested in a recirculating loop set-up, Jacobs and Edwards [107] concluded that the presence of sulfides in tap water increase corrosion rates by about one order (at pH 6.5) and two orders of magnitude(at pH 9.2) and passivation does not occur even after nine months of exposure. Additionally, electrochemical impedance spectroscopy (EIS), has been used to study the formation and dissolution of protective films (e.g., tenorite and cuprite), in presence of orthophosphate and chlorine [108], pH, DIC, and chloride [47]. Electrochemical techniques can also help elucidating the mechanism of copper corrosion. Polarization scans along with EIS have shown that corrosion is limited by the diffusion of copper ions through a cuprite film [65]. This conclusion has been further proved by several different studies using EIS [66,67,109]. Webster et al. [6] studied copper coupons using EIS at pH 8 and 6.8; and concluded that the oxide films at pH 8 were better represented by thin and compact films and at pH 6.8 they were porous and thicker. The study also applied EIS to copper coupons with microorganisms at pH 8, and concluded that the oxide films were similar to the ones at pH 6.8 instead of the ones at pH 8. This suggested that biofilms on copper were creating an acid microenvironment. The use of EIS to study copper coupons typically needs to adjust an equivalent electric system; however, there is no consensus on what system provides a better representation. Most studies choose a different electrical system [6,62,65,109], which makes the comparison of EIS results difficult.

Polarization scans may be used to understand the differences of copper corrosion at micro and macro scales [111]. Polarization scans can also suggest when copper is susceptible to pitting and what corrosion by-products are formed [112]. Recently, polarization scans have been used to study the persistence of corrosion inhibitors in copper pipes with tap water [113]. Finally, a new study using in situ Atomic Emission Spectroelectrochemistry (AESEC) [49] combining polarization with spectroscopy to measure copper ions in situ while a copper coupon was anodically polarized. This allowed measuring the difference between the copper released and the applied current, providing further understanding of the dissolution mechanism of copper, which was shown on Section 3.1. Therefore, electrochemical techniques and its combination with other techniques prove to be useful tools to understand copper corrosion.

### 4.3. Surface Characterization Techniques

Scanning electron microscopy (SEM) is a widely-used technique for surface characterization, including copper corrosion [102,107,114,115] and MIC studies [4,15,85,116]. As mentioned earlier, copper mobility in tap water depends on the reactive surface formed on the inner surface of the pipe, especially in the presence of a biofilm. Combining SEM and spectroscopic techniques it is possible to identify different configurations of solid corrosion by-products under different conditions. The morphology and structure of predominant inorganic passive films in copper pipes, identified as malachite, have different arrangements under MIC compared to abiotic corrosion [15,102]. The effect of microorganisms on the surface of copper pipes is shown in Figure 2b,c This may have implications in terms of the stability of the corrosion scale, particle detachment, and the development of homogeneous layers of inorganic compounds that passivate and protect the surface. 

Atomic force spectroscopy (AFS) uses atomic force microscopy (AFM) operating in force mode, offering visualization (images) and measurements of forces between the AFM tip and the sample [90]. With this technique, Beech and Summer [3] studied the association between the EPS secreted by cells and the iron sulfide (FeS) particles formed due to the effect of a marine biofilm formed on the surface of AISI 316 stainless steel. However, there is poor information about the application of this technique in MIC events in drinking water systems, especially in copper pipes. 

Structural identification of solid by-products formed at the solid–water interface is a further step to elucidate the mechanisms that govern copper corrosion [57,63]. For over four decades, powder X-ray diffraction (XRD) has been the technique of choice to identify the solid corrosion by-products formed on the inner-surface of copper pipes in contact with real and synthetic water [14,70,99,100,117,118]. After 30 h of stagnation in a copper pipe, dissolved copper concentration in the bulk water appears to follow the solubility of cupric hydroxide, tenorite, or malachite [14,57,63]. A condensed list of copper by-products identified by XRD is presented in Table 2. A more extensive review is available elsewhere [2].

Corrosion by-products in drinking water pipes are expected to be highly hydrated and with short-range ordering, rendering traditional XRD inadequate for their structural identification [48], even if the hydrated corrosion scale is measured in situ [119]. XRD is not element specific, and sample preparation may lead to artifacts, including dehydration and oxidation of the corrosion scale [14]. Efforts to overcome the limitations of XRD included the use of spectroscopic techniques, such as electron microprobe [120,121], XPS [97,122], and vibrational spectroscopy [123,124]. Often, these techniques are used in combination to provide a robust interpretation of the results of each individual technique. Electron microprobe analysis is an excellent tool to probe the chemical composition of the corrosion scale, while spectroscopic techniques can provide insights about the identity of the corrosion by-products.

X-ray absorption spectroscopy (XAS) offers several advantages to characterize the structural identity of corrosion by-products in drinking water pipes: it allows to probe the coordination chemistry of a single element of interest, it does not require sample dehydration, samples may have short or long-range ordering, and minimal sample quantities are needed to achieve an absorption signal (in the order of parts per million). Previous uses of XAS in corrosion studies include the study of imidazole as a potential copper chelating agent from EPS during episodes of MIC [125], and the determination of the coordination chemistry of copper in a malachite scale formed on the brass surface of a drinking water distribution system [15,102,126]. XAS has been used to test the nature of a corrosion scale formed under a MIC event [15], and cupric hydroxide was identified as one of the main components of the scale. However, systematic identification of corrosion solid by-products under controlled conditions (including water chemistry, and pipe ageing time) is still needed to support the role of cupric hydroxide in current corrosion theory. A brief list of techniques commonly used to study the corrosion solid by-products is presented in Table 3.

### 4.4. Techniques for Determination of Microbial Biofilm Populations

As in natural environments, biofilm formation in distribution systems is dynamic and typically involves diverse microbial populations following ecological succession, from the initial individual attachment to the final consolidation of a complex mature structure [133]. The synergic corrosion mechanisms described before depend on the activity of the microbial community, and the specific effects of different bacterial species may be important when certain groups dominate the biofilm. Keevil et al. [4] proposed a conceptual model for the biofilm colonization in copper pipes as a function of the spatial and chemical heterogeneity that produces different niches. Some copper resistant microorganisms may have pioneer functions by giving structural support, some others may interact with the initial colonizers establishing cooperative and competitive interactions, and as microenvironments develop, separated metabolic groups may distribute along the biofilm (e.g., aerobic heterotrophs, microaerophiles, anaerobes, and autotrophs). Thus, it is important to determine the biofilm microbial groups and their possible role in MIC. 

Modern molecular techniques allow the detection of environmental microorganisms as its culturability is very low [134]. However, most studies describing biofilm bacterial communities in corroded copper plumbing have used culture-dependent methods identifying aerobic heterotrophic bacteria involved in MIC [82,83,84,135]. An important limitation for carrying out culture-independent analyses in these systems may be the high amount of copper released when biofilms are extracted. Copper, among other heavy metals, could strongly inhibit DNA isolation and polymerase chain reaction (PCR). In other materials such as steel and concrete, the combination of traditional cultivation and molecular techniques, including cloning-sequencing of 16S rRNA PCR amplicons and fluorescent in situ hybridization (FISH), have shown an important presence at the solid–liquid interface of sulfate-reducing bacteria (SRB) and enterobacteria [136], methanogens [137], and sulfur-oxidizing bacteria (SOB) [138] in MIC events. From the identification of these functional groups, it has been demonstrated that the generation of reactive sulfur species by SRB (e.g., sulfides) and acid metabolites by SOB (e.g., sulfuric acid) can break the protective passivation layers and initiate corrosion. Using cultured SRB isolated from the sea on copper coupons made similar conclusions. The study showed that tested SRB are copper resistant and that their metabolism can enhance copper corrosion [139].

Microbial specific activity within the biofilm and corrosion scale matrix is also an area of interest that is suitable to be profiled with molecular tools such as quantitative PCR and DNA microarrays. In copper plumbing biofilms, the use of molecular markers targeting specific functions and the activity of enzymes (e.g., catalases and hydrogenases) or metabolites related to corrosion, the identification of copper resistance features, and the characterization of the properties of the biofilm EPS components, constitute next steps for the determination of microbial communities involved in copper plumbing corrosion. A newer culture independent approach to study microorganism activity in plumbing systems is the analysis of the metabolic profile of the water. This can be done using liquid chromatography–mass spectroscopy (LC-MS), gas chromatography–mass spectroscopy (GC-MS) and Dimensional Excitation/Emission (3DEEM) fluorescence spectroscopy of the water to identify the metabolites present in the water [140,141,142]. The authors suggest that this technique can be used to assess if the MIC is at an early or late stage and thus manage the problem accordingly, although further research is needed to identify the key metabolites.

## 5. Hydrodynamic Considerations

Corrosion in copper pipes from drinking water systems is a complex phenomenon that depends on a great number of variables related to water quality and operational conditions [2]. These variables combine into a series of physicochemical processes such as oxidation-reduction, acid–base equilibrium, precipitation-dissolution reactions, transport by diffusion, complexation of copper with organic compounds, etc. Operational conditions are an important factor that contributes to the corrosion rates and the amount of copper released into the water. In drinking water systems, pipes are subject to cycles of stagnation and flow. As mentioned earlier, the German regulation for testing corrosion in drinking water pipes DIN 50931-1 [143] states that, in a standard household water system, water remains stagnant 97% of the time and only 3% of the time corresponds to flow conditions. However, this pattern depends on local habits and generalization is difficult for other places. For instance, in Santiago de Chile, a study of consumption patterns revealed that the average stagnation time is 7.5 h [144].

### 5.1. Hydrodynamical Effects

During stagnation, dissolved chemical species are subject to diffusive transport. This mechanism is due to the chemical concentration gradient produced by the release of copper ions from the metal surface into the water. Even though diffusion transport remains during flow, it is small compared with convective transport. In this case, mass transport is mainly due to the velocity field within the pipe when water is flowing. Generally, plug flow is assumed when there is flow within a pipe [144], something that is not correct, especially at the metal–liquid interface where the hydrodynamic mechanisms play an important role on copper release. At this interface, advection poses chemical (changes in water chemical composition) and mechanical (shear stress due to water viscosity) effects. The literature reports on the connection between the hydrodynamic mechanisms and ion release from the metal surface, mainly due to mechanical abrasion of the protective oxide surface [7,145]. These assumptions can be adequate when the inner surface of the pipe is covered with abiotic corrosion products that are compact and of low porosity. However, they are inadequate when the pipe is covered with reactive microstructures, porous and soft, such as the ones found when the corrosion occurs in the presence of biofilms. When flow is established within a pipe, the spatial distribution of velocity generates changes in the water composition due to convective transport and also to shear stresses on surface of the pipe. A combination of processes such as desorption of weakly bound copper and detachment of biofilm pieces from the surface of the pipe can increase the mass flux of copper into the water. There is evidence of mass transfer at solid–liquid interfaces such as sediment-water [146,147,148]. Evidence shows that copper concentration under flow conditions can be an order of magnitude higher in the presence of bacteria, and that microorganisms changes the size of the particles released, suggesting the importance of hydrodynamic effects in the presence of a microbial biofilm [16].

### 5.2. Flushing Experiments

The study of the hydrodynamic effects on copper release in bio-corroded pipes has been analyzed by our group in field stagnation-flow [15] and laboratory experiments [149]. The field experimental system consisted of a well connected to a PVC pipe followed by 1 m of copper pipe with an internal diameter of 1.95 cm, and 300 mL of volume. Disinfection of the well water was done by a UV-radiation system located prior to the PVC connection. After 10 h of stagnation sequential water samples were taken from the tap to determine copper concentration until 11 L were extracted from the pipe. Flushing experiments were repeated over 10 consecutive days [15]. Laboratory experiments were performed in 1 m long pipes of 1.95 cm internal diameter. Jeria et al. [149] used new and aged (48 h and 1 week) pipes using water extracted from the field well. Aging was performed at a constant temperature (25 ± 1 °C). After aging, each pipe was kept stagnant during 8 h, followed by a period of constant flow. During flow, water samples were taken and a copper release curve was obtained. Two flow regimes were used in these experiments: laminar (Re < 1000) and transitional (2000 < Re < 4000). With this procedure, release curves were obtained considering five different combinations of aging and flow. 

Results obtained in laboratory and in field experiments with pipes affected by MIC suggest that assuming ideal plug-flow within the pipe underestimates the total mass released during flow. Although the curve of copper release decreases as the volume passing through the pipe increases, its shape does not agree with the release curve assuming ideal plug flow within the pipe. Indeed, according to experimental observations in the field, the average total mass of copper released was 8.1 mg, 9 times the mass obtained assuming plug flow [15]. This mass was obtained integrating the area below the curve of copper release with volume of water extracted for the 10 experiments performed. It varies between 6.1 and 12.6 mg with a standard deviation of 2 mg. For the situation assuming plug flow, the mass corresponds to the product of the pipe volume times the copper concentration at the end of the stagnation period. The ratio of released mass of copper observed experimentally and plug flow assumption was calculated for the pipe with biofilm (6.2 to 11.6) and for pipes without biofilm where this ratio varies between 1.1 and 2.0. For the pipes without biofilm, the mass of copper released varies from 0.09 to 0.16 mg. Mathematical modeling shows that the difference of copper release observed between pipes with and without biofilms can be explained by the fact that the biofilm acts as a copper reservoir of ions that can be released under flow conditions [17]. 

## 6. Conceptual Corrosion Model

In the following paragraphs, we review the mechanisms of copper corrosion in flow and stagnation, especially when corrosion occurs in the presence of a biofilm. Based on this review, we developed a conceptual model of copper corrosion using a mechanistic approach that divides the phenomenon into several processes that occur on different spatial and temporal scales [2,4,63,68,114,150,151]. The model is presented below in separate cases for stagnation and for flow.

### 6.1. Conceptual Model During Stagnation 

**Figure 5:**
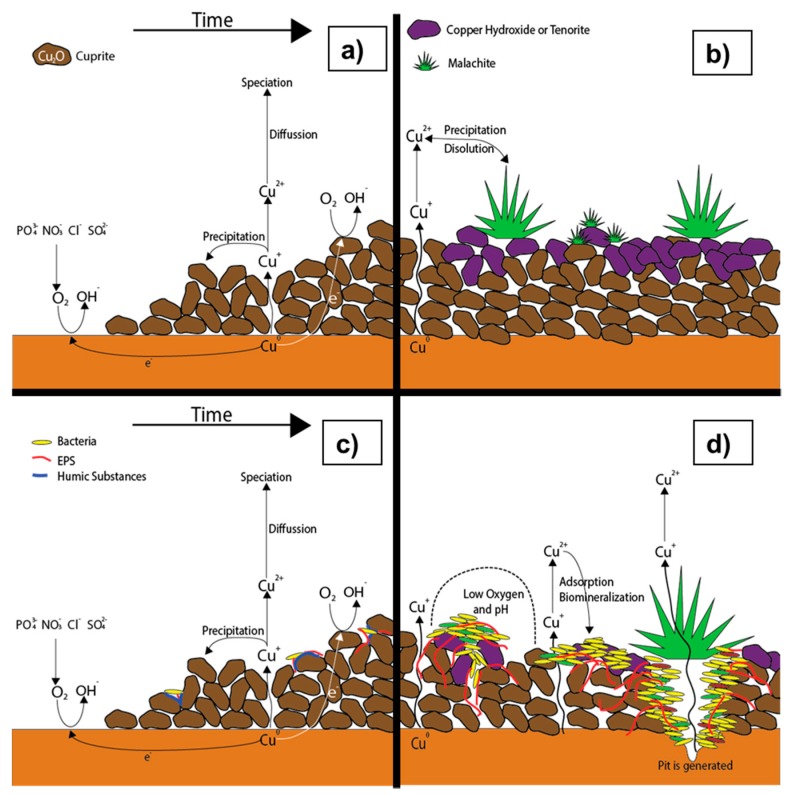
Conceptual corrosion model in copper pipes under stagnation. Abiotic conditions: (**a**) Within a few days, a porous and semiconducting cuprite layer that limits copper ion diffusion is formed, this process being the limiting step in corrosion. Anions present in the water competes with oxygen adsorption sites; (**b**) As cupric ions reach supersaturation, they precipitate forming more passivating scale, which can take months or years. Biotic conditions: (**c**) Humic substances adhere on the surface, letting bacteria grow; and (**d**) as time passes, the biofilm develops, creating low oxygen and pH environments and eventually pits that may cause pipe failure in a few years.

When a new pipe is in contact with aerated water, corrosion begins releasing copper ions to the water. These ions diffuse to the bulk water and speciate depending on water quality [13]. The first solid species to appear is a thin protective layer of cuprite growing rapidly on the inner wall of the pipe [2], the formation of this layer should involve precipitation and dissolution mechanisms [152]. This is a porous layer which limits the diffusion of copper ions, probably cuprous ions, through this film, making this process the limiting step on drinking water conditions [58,65]. The effect on the diffusion of copper ions depends on the porosity of this layer, which depends on the pH of the water, with higher porosity at lower pHs [153]. As time goes on, the cuprite the layer grows thicker limiting even more the corrosion, reaching a steady state at about 120 h at pH 7.6 [65], as shown on Figure 5a. Due to its semiconducting properties, the oxygen reduction occurs on the layer-liquid interface and it seems to be unaffected by the layer [2,65,99]. The absorption sites on which DO reduction occurs can be occupied by anions like chloride, sulfate, nitrate and phosphate [154]. 

As the oxygen reaction occurs, copper ions are released into water reaching supersaturation and thus precipitating into cupric scales, until equilibrium is reached. Generally speaking, solid corrosion by-products that are formed in the presence of drinking water are cuprite, tenorite, cupric hydroxide, and malachite [2,50,63,100,155]. As was mentioned before, the formation of cupric oxides can take months or years (Figure 5b). The effect of the scales is generally the passivation of the metal slowing even more the corrosion process [2,14,153]. Nevertheless, the effect of malachite is not clear. Merkel et al. claims it acts only as a reservoir of copper, without slowing the corrosion process [2]. On the other side, other authors claims malachite have a passivating effect on copper [14,102]. It is possible that it has different effects depending on the water physicochemical conditions, which can make the crystals grow in different forms, changing its effect on the surface. More research is needed in this area to elucidate the role of malachite on copper passivation. 

The fundamental process that allows for the release of copper in the presence of a biofilm is related to the formation of microenvironments caused by the biofilm spatial and chemical heterogeneousness on the pipe wall [4,156]. Several studies confirm the fact that bacterial colonies can move in and form colonies on the wall of drinking water distribution system pipes [4,74,82,84,155,157,158]. The environmental conditions inside a drinking water distribution pipe are such that colonies are formed mainly by aerobic heterotrophic micro-organisms that need organic carbon and oxygen for their metabolism [4]. 

Keevil [72] developed a conceptual model that determines the spatial and chemical heterogeneousness of biofilms on copper surfaces subjected to corrosion. According to this model, the biofilms formation is related to the pipe sorption of high molecular weight humic and fulvic substances, which create a thin film of nutrients that can lead to a complexation of Cu ions. This film is where the biofilm’s first cells attach (Figure 5c). First, copper decreases microbial number, when it is compared to other materials, but, as the biofilm develops, bacteria become resistant to copper and thus the bacteria increase [72,159]. As the number of cells begins to grow, the microorganisms start producing metabolic products and EPS, creating a matrix that agglomerates these cells and defends them from the Cu ions. As the biofilm begins to consolidate, a heterogeneous structure is generated and a diffusion layer is created so that in areas that are the furthest from the biofilm surface, a drop in nutrient concentration occurs, which can be seen by the results of heterogeneous biofilm mathematical models [160,161,162,163,164,165]. Additionally, this nutrient drop induces the formation of micro-channels in areas located furthest from the biofilm surface. 

The biofilm that forms on the copper surface is characterized for having a structure of few cells surrounded by abundant EPS, which are able to complexate copper ions at their carboxylic groups (COOH) [4,155,166]. The affinity between Cu ions and EPS, together with the biofilm heterogeneity are mechanisms that favor cuprosolvency, since Cu ions are removed from the metal surface in areas that are in direct contact with the biomass [155,158]. Another study used compounds similar to EPS and they found that copper was incorporated into these compounds, the following being most corrosive to the least corrosive: alginic acid, gum arabic, bacteria culture supernatant, and *Pseudomonas atlantica* EPS [167].

Another effect of the biofilm is the creation of microenvironments with low pH, making the cuprite layer more porous when it is under the biofilm influence [6], as shown on Figure 5d. This effect is obviously worse when the water is weakly buffered which is the typical situation when MIC occurs [135,168]. Along with the pH gradients, oxygen gradients are created due to nutrients consumption [169]. Thus, electrochemical micro-cells appear, where the anodic sites are located in the area under the biomass, and the cathodic sites on the surface that has no biomass, as presented in Figure 5d. As time passes, corrosion by-products are formed over the anodic zone, and a pit grows under the corrosion by-products [86]. It is hard to know when the pit is formed because its detection is normally when the pipe fails, which can happen in a few years [155].

Once the Cu^2+^ ions are formed, they are transported by diffusive mechanisms through the biofilm, as shown in Figure 5d. A small part of these ions precipitate, forming solid corrosion by-products in such a way that it causes a biomineralization of the biofilm [155]. Another fraction of ions undergoes complexation by EPS, and another fraction manages to pass through the biofilm, reaching the aqueous phase. Of this latter fraction, one part remains in the solution and is transported by diffusive mechanisms, forming different organic and inorganic aqueous species, and another part precipitates, forming or incorporating itself into the solid corrosion by-products on the pipe surface [63]. However, the physicochemical conditions that may exist in the interior of the pipe potentially cause the dissolution of these solid products, once again releasing copper into the aqueous phase.

The physicochemical mechanisms described above that produce the release of copper ions from the pipe surface are characterized at a microscopic spatial scale. The biofilm dimensions and the diffusion layer, which can reach a thickness of hundreds of microns, define this spatial scale. However, these ions continue to be transported by diffusive mechanisms, forming different aqueous species until reaching the core of the pipe. These last two mechanisms occur on a macroscopic scale that is defined by the pipe diameter, which is tens of millimeters wide. On a macroscopic scale, the diffusive transport elements depend on area/volume ratio of the pipe (area of the transversal section), as discussed in the literature [2].

Even though the ratio between the dimensions of the macroscopic and microscopic scales is approximately 100, the group of mechanisms that cause the release and transport of Cu works jointly, causing the released copper to be distributed along the pipe cross-section as long as the stagnation condition continues, a better visualization of the space scales is seen at the conceptual model developed by Pizarro et al. [17]. By globally analyzing the presence of solid corrosion by-products and heterogeneous biofilms on the pipe inner wall, it is possible to observe that these elements act like a reactive surface that controls the release of copper [15]. 

### 6.2. Conceptual Model During Flow

**Figure 6:**
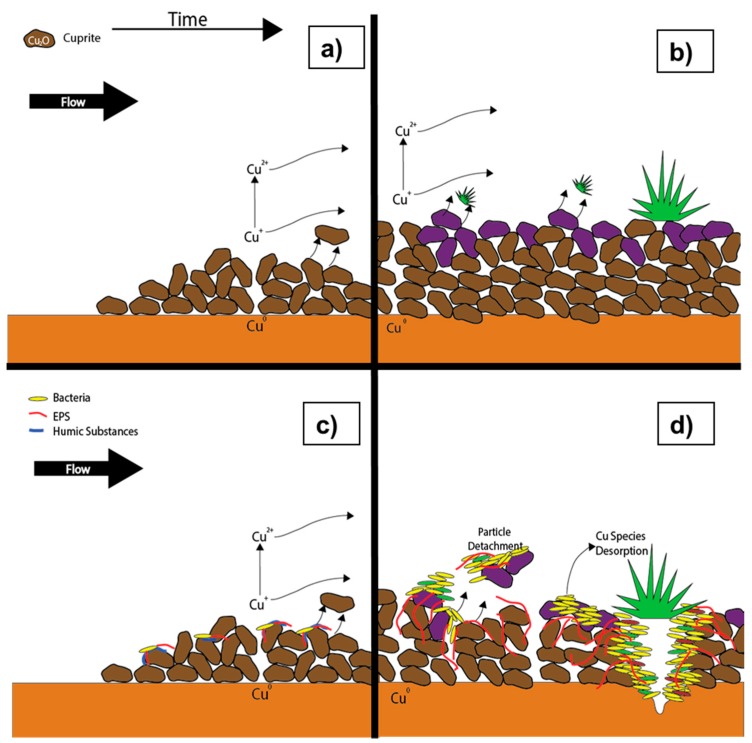
Conceptual corrosion model in copper pipes under flow. Abiotic conditions: (**a**) The cuprite layer growths thinner under permanent flow conditions, due to Cu(I) species flushing or shear stress; and (**b**) the flow produce the detachment of nanoparticles, probably malachite, increasing copper concentration. Biotic conditions: (**c**) Under permanent flow conditions there are more nutrients for the bacteria allowing them to grow faster; and (**d**) when the biofilm is developed, and a flow is imposed, the copper released is increased due to desorption of copper species and particle detachment.

During the flow condition, the velocity within the pipe generates chemical and physical mechanisms such as changes in the water composition due to convective transport and shear stress due to viscosity [15]. These mechanisms produce the desorption of weakly bound copper and the release of nanoparticles from the pipe inner surface, increasing the concentration of copper in the drinking water [70] as shown on Figure 6. It is unlikely that the release of particles is due to erosion corrosion, since it would require Reynolds numbers around three times larger than the ones found in typical household piping systems [16,170]. Feng et al. also showed that under constant flow conditions, the cuprite layer would grow as a thinner layer, due to a lower concentration at the surface of Cu(I) species needed for the cuprite nucleation or due to shear stress [65]. 

The literature mentions a connection between hydrodynamic mechanisms and the release of metal from the pipe surface, but principally oriented at mechanical abrasion during the flow condition [145,171]. However, when there is a reactive surface on the pipe surface wall that functions like a copper reservoir, it is released by mass transfer due to hydrodynamic effects [15]. An example of this reactive barrier are biofilms, they adsorb copper during stagnation and release it during flow, increasing the total amount of copper released [17]. 

The flow that circulates through the pipe during the flow condition determines the cross-sectional velocity profile. This profile has a parabolic form when the flow regime is laminar, in which viscous forces predominate over inertia. However, if the forces of inertia predominate, the flow is turbulent and the velocity profile has a logarithmic shape [17]. In terms of the duration of flow conditions in premise plumbing, it generally lasts for minutes, meaning it lasts for much less time than the stagnation condition [143,144]. However, this time is sufficient for extracting from the pipe the copper that was released, during stagnation, making the electrochemical reactions negligible during flow. Another important effect of the flow is the renewal of oxygen and nutrients that were consumed during stagnation (Figure 6). A study showed when a constant flow is imposed, a faster velocity flow produces more biofilm growth, this is probably because a faster velocity means more nutrients are available for the bacteria [172]. Thus, the renewal of the nutrients a key factor on the biofilm development.

A more recent study shows that under abiotic and biotic conditions the total amount of copper released when different flow velocities are applied does not change, but it defines the time at which copper release occurs [16]. It also shows that under biotic conditions, the released copper is around an order of magnitude higher [16], this increase could be explained by a higher amount of copper by-products release [89] as seen on Figure 6d. The size of these particles released depends on the Reynolds number, with higher Reynolds number bigger particles are released. In contrast, in abiotic conditions, no difference on the particle size was noticed with different Reynolds number [16]. 

### 6.3. Mathematical Modeling

Corrosion in copper pipes is divided in three general processes: oxidation-reduction processes, chemical reactions without electron transfer, and transport processes [4,63,68,114,150,151]. These occur simultaneously on different spatial and temporal scales, and include elements in solid and dissolved phases [2]. The oxidation-reduction reactions and reactions without electron transfer that happen in the aqueous corrosion-causing phase generally occur at intervals of seconds or minutes. The formation of oxides, however, is a process that can take hours and even days, and the accumulation of these oxides on the pipe surface can take from months to years [2]. This variability between the temporal scales of these processes may limit the information that can be obtained about the kinetics of some of these reactions in short-term experiments. In some cases, the combination of short-term laboratory experiments with a compilation of information obtained in real installations can help to better understand the processes and better interpret the results obtained in the laboratory. Furthermore, the inclusion of a specific process in a mathematical or conceptual corrosion model is a function of the temporal scenario defined for the model and of the temporal scale of this process.

Most of the mathematical corrosion models consists of using empirical relationships obtained from experimental observations of a process that is influenced by a series of parameters [2]. However, the comparison of experimental results must be carefully evaluated because of the variability of each study’s particular conditions, like the area/volume relationship (A/V, surface area of exposed copper to water/volume of water in the pipe), temperature, operating conditions, hydraulic regime, etc. For example, the literature reports on experiments carried out in conditions of stagnation and constant laminar flow (Re = 910) [63], and others in conditions of only stagnation [13]. The pipes that are normally used in household drinking water systems have a nominal diameter of ½ inch and ¾ inch, meaning that the A/V relationship varies from 200 to 300 m^−1^. In some electrochemical and MIC studies, coupons that have been used provide a small A/V relationship (A/V ~ 0.1 m^−1^), as they are oriented to analyze the processes at the metal–liquid interface [2,74,83]. However, in other electrochemical studies [62,108], A/V relationships of between 35 and 50 m^−1^ have been used so that results can be extrapolated to real pipes that are used in the distribution of drinking water. 

The first modeling approach was used to quantitatively estimate the quantity of copper released and consisted of using the layer of oxides on the pipe as a register of the corrosive attacks to which the pipe has been subject during its use [2,13,100]. This approach makes it possible to use thermodynamic equilibrium equations together with water quality and thermodynamic constant parameters, considering that all species are in equilibrium with a solid phase. Edwards et al. [13] used this approach to obtain equations that predict the copper released into a new pipe after a prolonged period of stagnation as a function of total alkalinity and pH. In this case, it was empirically determined that the solid that controls the solubility is cupric hydroxide. The choice of the solid phase with which species are balanced directly depends on the composition of the layer of oxides, which is a function of the pipe age. With this approach, Lagos et al. [100] developed equations to predict the release of copper in pipes of different ages as a function of pH and the concentrations of HCO_3_^−^ and SO_4_^2−^. In this study, dissolved copper measurements were taken in pipes of different ages after 8 h of stagnation. The equations obtained adequately reproduce the experimental observations noting that as the pipe age increases, the solid that controls the solubility varies according to the following sequence: langite, cupric hydroxide, azurite, broncantite, tenorite, and malachite [100]. Even though these models can sometimes provide reasonable estimations of copper released at the end of the stagnation time, they do not incorporate the influence of parameters like the A/V relationship or diffusion transport processes that occur during stagnation. 

Based on the stagnation curves obtained experimentally, Merkel et al. [2,114] developed a semi-empirical mathematical model that estimates the quantity of copper released and the consumption of oxygen in a pipe as a function of the stagnation time. Taxen et al. developed a more complex mechanistic model of copper release under stagnation [154]. It considers water composition and temperature, stagnation time, diameter, aging time and water use patterns as inputs. The processes considered in this model are copper speciation, diffusion, oxygen consumption, nucleation and crystal growth, adsorption processes that interferes with scale formation and natural organic matter (NOM) complexation. The model assumed that homogeneous reactions are in equilibrium and that solid liquid reactions are kinetically controlled, the model also considers that chloride, sulfate, nitrate, phosphate and NOM interferes with oxygen adsorption and scale formation making crystals grow “wrong”. This model successfully predicts the copper release of pipes with different water qualities. Even though this model is mechanistic, it does not include electrochemical reactions; it does not consider the cuprous ion and hydrodynamic effects; and the number of adjusted parameters makes it difficult to verify the robustness of the conceptual model. A recent study developed a model of MIC of copper [17]. The model considers processes occurring at different space and time scales, these processes are copper release under the biofilm, speciation of copper, diffusion of species, oxygen consumption, biofilm growth, copper complexation by the biofilm and hydrodynamic effects, but it does not electrochemical reactions. The model showed that the copper release curves under the presence of microorganisms in flow conditions can be explained by biofilms acting as a reservoir of copper, that release ions when the water is flowing. 

Other types of mathematical models that have been developed are oriented at similar processes that happen at the metal–liquid interface on a microscopic scale [169,173]. Malki and Baroux [173] developed a model to simulate the formation of pits on the surface of metal using a type of Monte Carlo and cellular automation (CA) simulation. Even though the model produces pits that are shaped similarly to those observed in real pipes, it did not include physical parameters that determine the shape and size of the pits. However, it is possible to use stochastic simulation methodologies to model processes related with corrosion. Another model of pitting corrosion in copper was developed by Ha et al. [174]. It is a mechanistic model that requires water quality parameters, temperature, electric potential, and pit depth, and it gives the pitting current, concentration of dissolved species and distribution of solid species through the pit.

Picioreanu and van Loosdrecht [62] developed a mathematical model of microscopic scale MIC on iron that includes the formation of electrochemical microcells due to the presence of a heterogeneous biofilm. The model determines the distribution of anodic and cathodic sites as a function of the gradients of oxygen and pH within the domain. Ion release was simulated by using a Butler-Volmer kinetic [175]. The model results are the spatial distribution of chemical species in the area adjacent to the biofilm for the stationary state. Even though the model was developed to simulate MIC on iron, an interesting approach appears here that could be applied to corrosion in copper pipes since it simulates processes that happen on a microscopic scale. However, the high quantity of electrochemical parameters that the model requires and the degree of variability in the magnitude of these parameters in the literature suggest that the results must be carefully interpreted and used as an orientation to understand the system behavior under certain conditions [169].

The mathematical models for corrosion of copper pipes that are currently available can reproduce the release of copper under different conditions. These models give us a better understanding of copper corrosion and the processes governing it. Nevertheless, there is still room for making improvements to the models by introducing: electrochemical reactions, interaction between scales and electrochemical reactions, migration processes, cuprous ions and species effect, growth of cuprite, detachment of particles, and turbulent flows.

## 7. Future Challenges

### 7.1. Early Detection of Corrosion and Biocorrosion

The detection of localized corrosion in copper pipes typically occurs when the pipe is already perforated by a pit, generating a leak. At this point, the only solution is to replace the pipe, thus the prevention is preferred. A prevention method is the addition of orthophosphate to the water which has been proved successful [19]. Another approach is to analyze the water quality parameters and predict if the water is susceptible to originate localized corrosion, by estimating the pitting potential of the pipe [112]. However, pitting corrosion on copper premise plumbing cannot be accurately predicted yet. 

There is a lack of techniques to detect the early presence of microorganisms, which induce corrosion on copper premise plumbing. To detect microorganisms in premise plumbing, it is required first a suspicion, either because a pinhole if found or blue water phenomenon is present. Then to confirm the formation of microbial biofilms it is necessary to analyze the inner pipe. Microorganisms typically found in copper pipes affected by biocorrosion, such as *Pseudomonas* sp. [83,155] and *Variovorax* sp. [85,116], have been used as model microorganisms to describe this phenomenon. However, in real life scenarios corrosion may be induced by the interaction of multiple bacteria interacting within a biofilm. Sometimes this interactions may induce surface passivation, inhibiting corrosion [95]. Thus, biocorrosion cannot be predicted by bacteria identification only. A possible approach for an early detection of biocorrosion events is the use of omics-based techniques to detect both bacteria and metabolisms occurring within the pipes. A combination of these techniques can be used for further understanding of the problem [142], and may lead to the prevention of biocorrosion.

### 7.2. Quantification of the Problem Extension

Abiotic and biotic reports of copper pinholes have been found all around the world. Its frequency was studied in the USA and it was found that there were hot spots with a higher frequency of pinhole leaks [18]. The frequency of copper pinholes in other countries has not been studied. It should be higher in countries with less strict water quality standards. 

High copper concentration in water is hard to detect by the consumer unless the water changes its color. If the concentration of copper is high enough to produce nausea or cramping, the consumer will probably avoid drinking it without knowing the cause. Thus, the extension of high copper concentration is not well known; especially in zones where the water quality is not well controlled such as rural areas. 

MIC has been reported in different locations around the world. The detection of MIC is not easily achieved and sometimes is not even considered on pitting cases; thus, it is hard to know the extension of MIC on copper premise plumbing.

### 7.3. Mathematical Modelling

There are several mathematical models of copper corrosion on premise copper pipes, however there is still room for improvement. None of the models predict the growth and dissolution of the passive layers on premise copper pipes, thus they do not predict when the passive layers are broken generating pitting. There are several models that explain and predict the growth of the passive layers on metals, such, Cabrera Mott, Felner Mott, PDM, “generalized model” [176], however they have not been applied to copper premise plumbing.

Biofilms on copper pipes generate microenvironments that are hard or impossible to characterize if they are embedded within corrosion by-products. Mathematical models could be used to predict the concentration and potential gradients generated by the biofilms. If this is coupled with a model that predicts the growth and dissolution layers, a better understanding of how microorganisms produce pitting could be achieved.

## 8. Conclusions

Usually, corrosion has been analyzed from the point of view of the materials and their associated deterioration, determining costs of replacement and maintenance. There is also an alternative view, from the water quality perspective, in which the corrosion is viewed as a source of dissolved species that will be incorporated into the water. The understanding of the processes that trigger corrosion in drinking water pipes, and the subsequent mechanisms of transport of copper to the water are of paramount importance to determine the final water quality and hence the effects on human health.

Copper corrosion and the subsequent release of Cu ions to the water is a complex phenomenon that consists of at least of three processes. First, one of the processes that enhance corrosion is biofilm formation. Biofilms create microenvironments that may influence corrosion. Second, biofilms act as a reactive layer, complexing ions that can be released when the conditions of the water change. Lastly, fluid flow is also an important factor since it can change environmental conditions at the solid–liquid interface, which releases the stored copper at the reactive layers. Fluid flow also exerts mechanical stress due to viscosity. This stress can detach pieces of the corrosion by-products or even pieces of the biofilms growing on the surface, increasing the concentration of copper in the water. The particles detached can be of small size (nanoparticles) that can be considered dissolved copper when using standard analytical techniques.

Modeling seems a promising tool for conceptualizing the processes occurring within the pipes and helps on the design of new experiments to test hypothesis. Further work is required to elucidate the relative importance of the processes highlighted previously.

## Figures and Tables

**Table 1:** Taste threshold concentrations of copper determined in distilled and drinking water.

Study	Country	Metallic Taste (mg/L of Cu)	
		(In Distilled Water)	(In Tap Water)
Cohen et al. [45] *	U.S.	6.6 ***	13 **
Zacarias et al. [46] *	Chile	2.5	2.6
Dietrich et al. [10]	U.S.	0.5	

(*) Defined as when 50% of the subjects could detect the taste; (**) In spring water with 200 mg/L alkalinity; (***) Water containing only 0.2 mg/L Cu and 0.13 mg/L Zn.

**Table 2:** Copper solid by-products of interest detected by X-ray Diffraction (XRD) in corrosion episodes.

By-Product	Chemical Formula	References
cuprite	Cu_2_O	[14,98,99,100,117,119]
tenorite	CuO	[14,99,117]
malachite	Cu_2_(OH)_2_CO_3_	[14,99,100,117]
cuprous chloride	CuCl	[97,99,119]
cupric chloride	CuCl_2_	[14,98]
atacamite	Cu_2_Cl(OH)_3_	[97,98,99]
antlerite	Cu_3_SO_4_(OH)_4_	[127]
brochantite	Cu_4_SO_4_(OH)_6_	[97]
posnjakite	Cu_4_(OH)_6_SO_4_·H_2_O	[14]
langite	Cu_4_(OH)_6_ SO_4_·2H_2_O	[100]

**Table 3:** Summary of available techniques to study the identity and composition of solid by-products formed during copper corrosion episodes.

Technique	Information	References
X-ray diffraction (XRD)	Crystalline identity of the corrosion scale. Requires certain knowledge about the chemical composition of the scale.	(See Table 2 for References)
Quartz crystal microbalance (QCM)	Evolution of the corrosion scale mass as a result of by-product deposition.	[123,128]
Time-of-Flight Secondary Ion Mass Spectroscopy (TOF-SIMS)	Atomic and molecular structure of organic and inorganic components of the scale.	[129,130]
X-ray Spectroscopy		
Energy dispersive X-ray spectroscopy (EDS)	Chemical composition of the corrosion scale. Often coupled with electron microscopy (EM).	[120,121]
X-ray photoelectron spectroscopy (XPS)	Chemical composition and speciation in the corrosion scale.	[97,122,131]
X-ray absorption spectroscopy (XAS)	Coordination chemistry and short-range ordering of a given element inside the corrosion scale.	[15,102,126]
Vibrational Spectroscopy		
Fourier transformed Infrared spectroscopy (FT-IR)	Molecular structure of organic and inorganic components of the scale.	[123,128]
Raman spectroscopy	Molecular structure of organic and inorganic components in the scale.	[124,132]
Electrochemical techniques		
Cyclic voltammetry	Scale composition based on its electrochemical properties.	[131]
Linear sweep voltammetry	Short-term experiment of accelerated pitting corrosion under different solutions.	[124]
Electrochemical impedance spectroscopy (EIS)	Electrochemical characterization and stability of formed films.	[47,122]

## References

[1] Shannon M.A., Bohn P.W., Elimelech M., Georgiadis J.G., Marinas B.J., Mayes A.M. (2008). Science and technology for water purification in the coming decades. Nature.

[2] Merkel T.H., Pehkonen S.O. (2006). General corrosion of copper in domestic drinking water installations: Scientific background and mechanistic understanding. Corros. Eng. Sci. Technol..

[3] Beech W.B., Sunner J. (2004). Biocorrosion: Towards understanding interactions between biofilms and metals. Curr. Opin. Biotechnol..

[4] Keevil C.W. (2004). The physico-chemistry of biofilm-mediated pitting corrosion of copper pipe supplying potable water. Water Sci. Technol..

[5] Walker J.T., Dowsett A.B., Dennis P.J.L., Keevil C.W. (1991). Continuous culture studies of biofilm associated with copper corrosion. Int. Biodeterior..

[6] Webster B.J., Werner S.E., Wells D.B., Bremer P.J. (2000). Microbiologically influenced corrosion of copper in potable water systems—pH effects. Corrosion.

[7] Oliphant R.J. (2003). Causes of Copper Corrosion in Plumbing Systems.

[8] APHA, AWWA, WEF (2005). Standard Methods for the Examination of Water and Wastewater.

[9] Zietz B.P., Dieter H.H., Lakomek M., Schneider H., Kessler-Gaedtke B., Dunkelberg H. (2003). Epidemiological investigation on chronic copper toxicity to children exposed via the public drinking water supply. Sci. Total Environ..

[10] Dietrich A.M., Glindemann D., Pizarro F., Gidi V., Olivares M., Araya M., Camper A., Duncan S., Dwyer S., Whelton A.J. (2004). Health and aesthetic impacts of copper corrosion on drinking water. Water Sci. Technol..

[11] Xu P., Huang S., Wang Z., Lagos G. (2006). Daily intakes of copper, zinc and arsenic in drinking water by population of Shanghai, China. Sci. Total Environ..

[12] Edwards M., Jacobs S., Taylor R.J. (2000). The blue water phenomenon. J. Am. Water Works Assoc..

[13] Edwards M., Schock M.R., Meyer T.E. (1996). Alkalinity, pH, and copper corrosion by-product release. J. Am. Water Works Assoc..

[14] Schock M.R., Lytle D.A., Clement J.A. (1995). Effect of pH, DIC, Ortophosphaste and Sulphate on Cuprosolvency.

[15] Calle G.R., Vargas I.T., Alsina M.A., Pasten P.A., Pizarro G.E. (2007). Enhanced copper release from pipes by alternating stagnation and flow events. Environ. Sci. Technol..

[16] Olivares T.E., Cienfuegos R., Vargas I.T., Pizarro G.E. (2014). Experimental evidence for enhanced copper release from domestic copper plumbing under hydrodynamic control. Corros. Sci..

[17] Pizarro G.E., Vargas I.T., Pasten P.A., Calle G.R. (2014). Modeling mic copper release from drinking water pipes. Bioelectrochemistry.

[18] Farooqi O.E. (2006). An Assessment and Modeling of Copper Plumbing Pipe Failures Due to Pinhole Leaks. Master’s Thesis.

[19] Pinhole Leaks & Corrosion Control. https://www.wsscwater.com/water-quality--stewardship/research/pinhole-leaks--corrosion-control.html.

[20] Lytle D.A., Liggett J. (2016). Impact of water quality on chlorine demand of corroding copper. Water Res..

[21] Araya M., Pena C., Pizarro F., Olivares M. (2003). Gastric response to acute copper exposure. Sci. Total Environ..

[22] Araya M., Olivares M., Pizarro F., Llanos A., Figueroa G., Uauy R. (2004). Community-based randomized double-blind study of gastrointestinal effects and copper exposure in drinking water. Environ. Health Perspect..

[23] Furukawa N., Hatano M. (1998). An acute experiment on retrograde intestinal peristalsis with emesis using decerebrated dogs. J. Auton. Nerv. Syst..

[24] Reisman D., Peirano J., Lewis D., Basu D., Hohrseiter D. (1987). Summary Review of the Health Effects Associated with Copper: Health Issue Assessment.

[25] W.H.O. (2011). Guidelines for Drinking-Water Quality.

[26] Pettersson R., Rasmussen F. (1999). Daily intake of copper from drinking water among young children in Sweden. Environ. Health Perspect..

[27] Pandit A., Bhave S. (1996). Present interpretation of the role of copper in Indian childhood cirrhosis. Am. J. Clin. Nutr..

[28] Araya M., McGoldrick M.C., Klevay L.M., Strain J.J., Robson P., Nielsen F., Olivares M., Pizarro F., Johnson L., Poirier K.A. (2001). Determination of an acute no-observed-adverse-effect level (NOAEL) for copper in water. Regul. Toxicol. Pharmacol..

[29] Olivares M., Araya M., Pizarro F., Uauy R. (2001). Nausea threshold in apparently healthy individuals who drink fluids containing graded concentrations of copper. Regul. Toxicol. Pharmacol..

[30] Gotteland M., Araya M., Pizarro F., Olivares M. (2001). Effect of acute copper exposure on gastrointestinal permeability in healthy volunteers. Dig. Dis. Sci..

[31] Pizarro F., Olivares M., Uauy R., Contreras P., Rebelo A., Gidi V. (1999). Acute gastrointestinal effects of graded levels of copper in drinking water. Environ. Health Perspect..

[32] National research council (2000). Report on copper in drinking water. J. Environ. Health.

[33] Olivares M., Uauy R. (1996). Limits of metabolic tolerance to copper and biological basis for present recommendations and regulations. Am. J. Clin. Nutr..

[34] Pratt W.B., Omdahl J.L., Sorenson J. (1985). Lack of effects of copper gluconate supplementation. Am. J. Clin. Nutr..

[35] O’Donohue J., Reid M., Varghese A., Portmann B., Williams R. (1999). A case of adult chronic copper self-intoxication resulting in cirrhosis. Eur. J. Med. Res..

[36] Kitzberger R., Madl C., Ferenci P. (2005). Wilson disease. Metab. Brain Dis..

[37] Torsdottir G., Kristinsson J., Sveinbjornsdottir S., Snaedal J., Johannesson T. (1999). Copper, ceruloplasmin, superoxide dismutase and iron parameters in parkinson’s disease. Pharmacol. Toxicol..

[38] Mercer J.F.B. (2001). The molecular basis of copper-transport diseases. Trends Mol. Med..

[39] Brown D.R., Kozlowski H. (2004). Biological inorganic and bioinorganic chemistry of neurodegeneration based on prion and alzheimer diseases. Dalton Trans..

[40] Khiari D., Bruchet A., Gittelman T., Matia L., Barrett S., Suffett I.H., Hund R. (1999). Distribution-generated taste-and-odor phenomena. Water Sci. Technol..

[41] Rigal S., Danjou J. (1999). Tastes and odors in drinking water distribution systems related to the use of synthetic materials. Water Sci. Technol..

[42] Dietrich A.M., Burlingame G.A., Vest C., Hopkins P. (2004). Rating method for evaluating distribution-system odors compared with a control. Water Sci. Technol..

[43] Suffet I.H.M., Corado A., Chou D., McGuire M.J., Butterworth S. (1996). Awwa taste and odor survey. J. Am. Water Work Assoc..

[44] Secondary Drinking Water Standards: Guidance for Nuisance Chemicals. https://www.epa.gov/dwstandardsregulations/secondary-drinking-water-standards-guidance-nuisance-chemicals.

[45] Cohen J.M., Kamphake L.J., Harris E.K., Woodward R.L. (1960). Taste threshold concentrations of metals in drinking water. J. Am. Water Work Assoc..

[46] Zacarías I., Yáñez C.G., Araya M., Oraka C., Olivares M., Uauy R. (2001). Determination of the taste threshold of copper in water. Chem. Senses.

[47] Palit A., Pehkonen S.O. (2000). Copper corrosion in distribution systems: Evaluation of a homogeneous Cu_2_O film and a natural corrosion scale as corrosion inhibitors. Corros. Sci..

[48] Boulay N., Edwards M. (2001). Role of temperature, chlorine, and organic matter in copper corrosion by-product release in soft water. Water Res..

[49] Zhou P., Hutchison M., Scully J., Ogle K. (2016). The anodic dissolution of copper alloys: Pure copper in synthetic tap water. Electrochim. Acta.

[50] Ives D.J., Rawson A.E. (1962). Copper corrosion III. Electrochemical theory of general corrosion. J. Electrochem. Soc..

[51] Ives D.J., Rawson A.E. (1962). Copper corrosion I. Thermodynamic aspects. J. Electrochem. Soc..

[52] Ives D.J., Rawson A.E. (1962). Copper corrosion II. Kinetic studies. J. Electrochem. Soc..

[53] Ives D.J.G., Rawson A.E. (1962). Copper corrosion IV. The effects of saline additions. J. Electrochem. Soc..

[54] Pourbaix M. (1974). Atlas of Electrochemical Equilibria in Aqueous Solutions.

[55] Cong H., Scully J.R. (2010). Effect of chlorine concentration on natural pitting of copper as a function of water chemistry. J. Electrochem. Soc..

[56] Atlas D., Coombs J., Zajicek O. (1982). The corrosion of copper by chlorinated drinking waters. Water Res..

[57] Edwards M., Powers K., Hidmi L., Schock M.R. (2001). The role of pipe ageing in copper corrosion by-product release. Water Sci. Techonl..

[58] Pehkonen S.O., Palit A., Zhang X. (2002). Effect of specific water quality parameters on copper corrosion. Corrosion.

[59] Hidmi L., Edwards M. (1999). Role of temperature and pH in Cu(OH)(2) solubility. Environ. Sci. Technol..

[60] Rushing J.C., Edwards M. (2004). The role of temperature gradients in residential copper pipe corrosion. Corros. Sci..

[61] Lagos G. (2001). Corrosion of Copper Plumbing Tubes and the Release of Copper by-Products to Drinking Water.

[62] Zhang X., Pehkonen S.O., Kocherginsky N., Andrew Ellis G. (2002). Copper corrosion in mildly alkaline water with the disinfectant monochloramine. Corros. Sci..

[63] Merkel T.H., Groß H.-J., Wernera W., Dahlkeb T., Reichertera S., Beuchleb G., Eberle S.H. (2002). Copper corrosion by-product release in long-term stagnation experiments. Water Res..

[64] Alex T., Johannsen K. (2000). Copper in drinking water supplies. The development of a kinetic model to describe the copper by-product release in corrosion tests using din 50931-1. VOM WASSER.

[65] Feng Y., Teo W.K., Siow K.S., Tan K.L., Hsieh A.K. (1996). The corrosion behaviour of copper in neutral tap water. Part I: Corrosion mechanisms. Corros. Sci..

[66] Metikos-Hukovic M., Babic R., Marinovic A. (1998). Spectrochemical characterization of benzotriazole on copper. J. Electrochem. Soc..

[67] Folquer M.E., Ribotta S.B., Real S.G., Gassa L.M. (2002). Study of copper dissolution and passivation processes by electrochemical impedance spectroscopy. Corrosion.

[68] Edwards M., Sprague N. (2001). Organic matter and copper corrosion by-product release: A mechanistic study. Corros. Sci..

[69] Geesey G.G., Bremer P.J., Videla H.A., Lewandowski Z., Lutey R.W. (1993). Interactions of exopolymers of corrosive biofilm microorganisms with copper ions. Proceedings of the NSF-CONICET Workshop on Biofouling and Biocorrosion: Metal/Microbe Interactions.

[70] Vargas I.T., Pavissich J.P., Olivares T.E., Jeria G.A., Cienfuegos R.A., Pasten P.A., Pizarro G.E. (2010). Increase of the concentration of dissolved copper in drinking water systems due to flow-induced nanoparticle release from surface corrosion by-products. Corros. Sci..

[71] Gagnon G.A., Rand J.L., O’Leary K.C., Rygel A.C., Chauret C., Andrews R.C. (2005). Disinfectant efficacy of chlorite and chlorine dioxide in drinking water biofilms. Water Res..

[72] Lehtola M.J., Miettinen K.T., Keinanen M.M., Kekki T.K., Laine O., Hirvonen A., Vartiainen T., Martikainen P.J. (2004). Microbiology, chemistry and biofilm development in a pilot drinking water distribution system with copper and plastic pipes. Water Res..

[73] Trevors J.T., Cotter C.M. (1990). Copper toxicity and uptake in microorganisms. J. Ind. Microbiol..

[74] Critchley M.M., Cromar N.J., McClure N., Fallowfield H.J. (2001). Biofilms and microbially influenced cuprosolvency in domestic copper plumbing systems. J Appl. Microbiol..

[75] Berry D., Xi C.W., Raskin L. (2006). Microbial ecology of drinking water distribution systems. Curr. Opin. Biotechnol..

[76] Lehtola M.J., Miettinen I.T., Lampola T., Hirvonen A., Vartiainen T., Martikainen P.J. (2005). Pipeline materials modify the effectiveness of disinfectants in drinking water distribution systems. Water Res..

[77] LeChevallier M.W., Babcock T.M., Lee R.G. (1987). Examination and characterization of distribution system biofilms. Appl. Environ. Microbiol..

[78] Flemming H.C. (2002). Biofouling in water systems—Cases, causes and countermeasures. Appl. Microbiol. Biotechnol..

[79] Costerton J.W., Stewart P.S., Greenberg E.P. (1999). Bacterial biofilms: A common cause of persistent infections. Science.

[80] Harrison J.J., Ceri H., Turner R.J. (2007). Multimetal resistance and tolerance in microbial biofilms. Nat. Rev. Microbiol..

[81] Flemming H.C., Neu T.R., Wozniak D.J. (2007). The eps matrix: The “house of biofilm cells”. J. Bacteriol..

[82] Critchley M.M., Pasetto R., O’Halloran R.J. (2004). Microbiological influences in ‘blue water’ copper corrosion. J. Appl. Microbiol..

[83] Critchley M.M., Cromar N.J., McClure N.C., Fallowfield H.J. (2003). The influence of the chemical composition of drinking water on cuprosolvency by biofilm bacteria. J. Appl. Microbiol..

[84] Dutkiewicz C., Fallowfield H.J. (1998). Assessment of microbial involvement in the elevation of copper levels in drinking water. J. Appl. Microbiol..

[85] Pavissich J.P., Vargas I.T., Gonzalez B., Pasten P.A., Pizarro G.E. (2010). Culture dependent and independent analyses of bacterial communities involved in copper plumbing corrosion. J. Appl. Microbiol..

[86] Burleigh T.D., Gierke C.G., Fredj N., Boston P.J. (2014). Copper tube pitting in santa fe municipal water caused by microbial induced corrosion. Materials.

[87] Siedlarek H., Wagner D., Fischer W., Paradies H. (1994). Microbiologically influenced corrosion of copper: The ionic transport properties of biopolymers. Corros. Sci..

[88] Letelier M.V., Lagos G.E., Reyes A. (2008). Chemical characterization of blue stains in domestic fixtures in contact with drinking water. Environ. Monit. Assess..

[89] Vargas I.T., Alsina M.A., Pavissich J.P., Jeria G.A., Pasten P.A., Walczak M., Pizarro G.E. (2014). Multi-technique approach to assess the effects of microbial biofilms involved in copper plumbing corrosion. Bioelectrochemistry.

[90] Beech I.B., Sunner J.A., Hiraoka K. (2005). Microbe-surface interactions in biofouling and biocorrosion processes. Int. Microbiol..

[91] Videla H.A., Herrera L.K. (2005). Microbiologically influenced corrosion: Looking to the future. Int. Microbiol..

[92] Little B.J., Wagner P.A., Lewandowski Z., Ribbe P.H. (1997). Spatial Relationships between Bacteria and Mineral Surfaces. Geomicrobiology: Interactions between Microbes and Minerals.

[93] Garcia F., Lopez A.L.R., Guillen J.C., Sandoval L.H., Gonzalez C.R., Castano V. (2012). Corrosion inhibition in copper by isolated bacteria. Anti-Corros. Methods Mater..

[94] Bremer P.J., Geesey G.G. (1991). Laboratory-based model of microbiologically induced corrosion of copper. Appl. Environ. Microbiol..

[95] Kip N., van Veen J.A. (2014). The dual role of microbes in corrosion. ISME J..

[96] Adeloju S.B., Hughes H.C. (1986). The corrosion of copper pipes in high chloride-low carbonate mains water. Corros. Sci..

[97] Mankowski G., Duthil J.P., Giusti A. (1997). The pit morphology on copper in chloride- and sulphate-containing solutions. Corros. Sci..

[98] Chmielova M., Seidlerova J., Weiss Z. (2003). X-ray diffraction phase analysis of crystalline copper corrosion products after treatment in different chloride solutions. Corros. Sci..

[99] Callot P., Jaegle A., Kalt A., Nanse G. (1978). Pitting corrosion of copper tubes and carbon deposits: Escs studies. Mater. Corros..

[100] Lagos G.E., Cuadrado C.A., Letelier M.V. (2001). Aging of copper pipes by drinking water. J. Am. Water Work Assoc..

[101] Le Gal La Salle A., Jardy A., Rosset R., Keddam M., Caramel A., Noel D. (1992). Copper corrosion, passivation and protection in aqueous solutions. I. Cyclic mechanism of the corrosion. Mem. Etud. Sci. Rev. Met..

[102] Vargas I.T., Alsina M.A., Pasten P.A., Pizarro G.E. (2009). Influence of solid corrosion by-products on the consumption of dissolved oxygen in copper pipes. Corrosion science. Corros. Sci..

[103] Eriksen R.S., Mackey D.J., van Dam R., Nowak B. (2001). Copper speciation and toxicity in macquarie harbour, tasmania: An investigation using a copper ion selective electrode. Mar. Chem..

[104] Carvallo M.J. (2005). Evaluación de la capacidad de Sorción de Cobre por Biomasa Usando Electrodo Ise: Implicaciones Para un Modelo de Biocorrosión de Cañerías de Cobre.

[105] Xia X., Xie C., Cai S., Yang Z., Yang X. (2006). Corrosion characteristics of copper microparticles and copper nanoparticles in distilled water. Corros. Sci..

[106] Pizarro F., Olivares M., Araya M., Gidi V., Uauy R. (2001). Gastrointestinal effects associated with soluble and insoluble copper in drinking water. Environ. Health Perspect..

[107] Jacobs S., Edwards M. (2000). Sulfide scale catalysis of copper corrosion. Water Res..

[108] Zhe Y., Pehkonen S.O. (2004). Copper corrosion kinetics and mechanisms in the presence of chlorine and orthophosphate. Water Sci. Technol..

[109] Feng Y., Teo W.K., Siow K.S., Hsieh A.K. (1996). The corrosion behaviour of copper in neutral tap water. Part II. Determination of corrosion rates. Corros. Sci..

[110] Valcarce M., De Sanchez S., Vazquez M. (2006). A comparative analysis of copper and brass surface films in contact with tap water. J. Mater. Sci..

[111] Arjmand F., Adriaens A. (2012). Influence of pH and chloride concentration on the corrosion behavior of unalloyed copper in NaCl solution: A comparative study between the micro and macro scales. Materials.

[112] Cong H.B., Michels H.T., Scully J.R. (2009). Passivity and pit stability behavior of copper as a function of selected water chemistry variables. J. Electrochem. Soc..

[113] Burstein G., Bi H., Kawaley G. (2016). The persistence of inhibition of copper corrosion in tap water. Electrochim. Acta.

[114] Merkel T.H. (2004). Copper corrosion: Understanding and modelling general corrosion. Water Sci. Technol..

[115] Vargas I.T., Alsina M.A., Pasten P.A., Pizarro G.E. (2008). Influence of malachite morphology on copper release in drinking water systems. Proceedings of the European Corrosion Congress (EUROCORR 2008).

[116] Reyes A., Letelier M.V., De la Iglesia R., Gonzalez B., Lagos G. (2008). Biologically induced corrosion of copper pipes in low-pH water. Int. Biodeterior. Biodegrad..

[117] Adeloju S.B., Duan Y.Y. (1994). Influence of bicarbonate ions on stability of copper oxides and copper pitting corrosion. Br. Corros. J..

[118] Vargas I.T., Pasten P.A., Pizarro G.E. (2010). Empirical model for dissolved oxygen depletion during corrosion of drinking water copper pipes. Corros. Sci..

[119] Sathiyanarayanan S., Sahre M., Kautek W. (1999). In-situ grazing incidence X-ray diffractometry observation of pitting corrosion of copper in chloride solutions. Corros. Sci..

[120] Fernandes P.J.L. (1998). Type I pitting of copper tubes from a water distribution system. Eng. Fail Anal..

[121] Srivastava A., Balasubramaniam R. (2005). Microstructural characterization of copper corrosion in aqueous and soil environments. Mater. Charact..

[122] Shim J.J., Kim J.G. (2004). Copper corrosion in potable water distribution systems: Influence of copper products on the corrosion behavior. Mater. Lett..

[123] Kautek W., Geuß M., Sahre M., Zhao P., Mirwald S. (1997). Multi-method analysis of the metal/electrolyte interface: Scanning force microscopy (SFM), quartz microbalance measurements (QMB), fourier transform infrared spectroscopy (FTIR) and grazing incidence X-ray diffractometry (GIXD) at a polycrystalline copper electrode. Surf. Interface Anal..

[124] Christy A.G., Lowe A., Otieno-Alego V., Stoll M., Webster R.D. (2004). Voltammetric and raman microspectroscopic studies on artificial copper pits grown in simulated potable water. J. Appl. Electrochem..

[125] Paradies H.H., Hänßel I., Fischer W., Wagner D. (1990). Microbiologically Induced Corrosion on Copper Pipes.

[126] Frenkel A.I., Korshin G.V. (1999). Exafs studies of the chemical state of lead and copper in corrosion products formed on the brass surface in potable water. J. Synchrotron Radiat..

[127] Watanabe M., Higashi Y., Tomita M., Ichino T. (2002). Microstructural analysis of artificially formed patinas on copper. Electrochem. Solid State Lett..

[128] Fonsati M., Zucchi F., Trabanelli G. (1998). Study of corrosion inhibition of copper in 0.1 m NaCl using the eqcm technique. Electrochim. Acta.

[129] Brusic V., Frisch M., Eldridge B., Novak F., Kaufman F., Rush B., Frankel G. (1991). Copper corrosion with and without inhibitors. J. Electrochem. Soc..

[130] Lewandowski B.R., Lytle D.A., Garno J.C. (2010). Nanoscale investigation of the impact of pH and orthophosphate on the corrosion of copper surfaces in water. Langmuir.

[131] Abelev E., Starosvetsky D., Ein-Eli Y. (2007). Potassium sorbate—A new aqueous copper corrosion inhibitor electrochemical and spectroscopic studies. Electrochim. Acta.

[132] Frost R.L. (2003). Raman spectroscopy of selected copper minerals of significance in corrosion. Spectrochim. Acta Mol. Biomol. Spectrosc..

[133] Martiny A.C., Jorgensen T.M., Albrechtsen H.J., Arvin E., Molin S. (2003). Long-term succession of structure and diversity of a biofilm formed in a model drinking water distribution system. Appl. Environ. Microbiol..

[134] Amann R.I., Ludwig W., Schleifer K.H. (1995). Phylogenetic identification and in-situ detection of individual microbial-cells without cultivation. Microbiol. Rev..

[135] Bremer P.J., Webster B.J., Wells D.B. (2001). Biocorrosion of copper in potable water. J. Am. Water Work Assoc..

[136] Bermont-Bouis D., Janvier M., Grimont P.A.D., Dupont I., Vallaeys T. (2007). Both sulfate-reducing bacteria and enterobacteriaceae take part in marine biocorrosion of carbon steel. J. Appl. Microbiol..

[137] Zhang T., Fang H.H.P., Ko B.C.B. (2003). Methanogen population in a marine biofilm corrosive to mild steel. Appl. Microbiol. Biotechnol..

[138] Hernandez M., Marchand E.A., Roberts D., Peccia J. (2002). In situ assessment of active thiobacillus species in corroding concrete sewers using fluorescent rna probes. Int. Biodeterior. Biodegrad..

[139] Chen S., Wang P., Zhang D. (2014). Corrosion behavior of copper under biofilm of sulfate-reducing bacteria. Corros. Sci..

[140] Beale D.J., Dunn M.S., Marney D. (2010). Application of gc-ms metabolic profiling to ‘blue-green water’ from microbial influenced corrosion in copper pipes. Corros. Sci..

[141] Beale D.J., Dunn M.S., Morrison P.D., Porter N.A., Marlow D.R. (2012). Characterisation of bulk water samples from copper pipes undergoing microbially influenced corrosion by diagnostic metabolomic profiling. Corros. Sci..

[142] Beale D.J., Karpe A.V., Jadhav S., Muster T.H., Palombo E.A. (2016). Omics-based approaches and their use in the assessment of microbial-influenced corrosion of metals. Corros. Rev..

[143] DIN (1999). Corrosion of Metals—Corrosion Testing of Drinking Water Distribution Systems—Part 1: Determining Changes to the Composition of Drinking Water.

[144] Lagos G.E., Maggi L.C., Peters D., Reveco F. (1999). Model for estimation of human exposure to copper in drinking water. Sci. Total Environ..

[145] Poulson B. (1993). Advances in understanding hydrodynamic effects on corrosion. Corros. Sci..

[146] Fries J.S. (2007). Predicting interfacial diffusion coefficients for fluxes across the sediment-water interface. J. Hydraul. Eng..

[147] Higashino M., Stefan H.G. (2004). Diffusive boundary layer development above a sediment-water interface. Water Environ. Res..

[148] Steinberger N., Hondzo M. (1999). Diffusional mass transfer at sediment-water interface. J. Environ. Eng..

[149] Jeria G.A., Vargas I.T., Walczak M.M., Pastén P.A., Pizarro G.E. Effect of hydrodynamic conditions on copper release in drinking water systems. Proceedings of the European Corrosion Congress (EUROCORR 2010).

[150] Harrison D.B., Nicholas D.M., Evans G.M. (2004). Pitting corrosion of copper tubes in soft drinking waters: Corrosion mechanism. J. Am. Water Work Assoc..

[151] Geesey G.G., Bremer P.J., Fischer W., Wagner D., Keevil C.W., Walker J.T., Chamberlain A.H.L., Angell P., Geesey G.G., Lewandowski Z., Flemming H.C. (1994). Unusual types of pitting corrosion of copper tubes in potable water systems. Biofouling and Biocorrosion in Industrial Water Systems.

[152] De Chialvo M.G., Zerbino J., Marchiano S., Arvia A. (1986). Correlation of electrochemical and ellipsometric data in relation to the kinetics and mechanism of Cu_2_O electroformation in alkaline solutions. J. Appl. Electrochem..

[153] Feng Y., Siow K.S., Teo W.K., Tan K.L., Hsieh A.K. (1997). Corrosion mechanisms and products of copper in aqueous solutions at various pH values. Corrosion.

[154] Taxen C., Letelier M.V., Lagos G. (2012). Model for estimation of copper release to drinking water from copper pipes. Corros. Sci..

[155] Wagner D., Chamberlain A.H.L. (1997). Microbiologically influenced copper corrosion in potable water with emphasis on practical relevance. Biodegradation.

[156] Battin T.J., Kaplan L.A., Newbold J.D., Hansen C.M.E. (2003). Contributions of microbial biofilms to ecosystem processes in stream mesocosms. Nature.

[157] Arens P., Tuschewitzki G.J., Wollmann H., Follner H., Jacobi H. (1995). Indicators for microbiologically induced corrosion of copper pipes in a cold water plumbing system. Zbl. Hyg. Umweltmed..

[158] Walker J.T., Wagner D., Fischer W., Keevil C.W. (1994). Rapid detection of biofilm on corroded copper pipes. Biofouling.

[159] Waines P.L., Moate R., Moody A.J., Allen M., Bradley G. (2011). The effect of material choice on biofilm formation in a model warm water distribution system. Biofouling.

[160] Noguera D.R., Pizarro G., Regan J.M., Ghannoum M.A., O’Toole G.A. (2004). Modeling biofilms. Microbial Biofilms.

[161] Xavier J.B., Picioreanu C., van Loosdrecht M.C.M. (2005). A framework for multidimensional modelling of activity and structure of multispecies biofilms. Environ. Microbiol..

[162] Picioreanu C., Kreft J.U., van Loosdrecht M.C.M. (2004). Particle-based multidimensional multispecies biofilm model. Appl. Environ. Microbiol..

[163] Chang I., Gilbert E.S., Eliashberg N., Keasling J.D. (2003). A three-dimensional, stochastic simulation of biofilm growth and transport-related factors that affect structure. Microbiology.

[164] Pizarro G., Griffeath D., Noguera D.R. (2001). Quantitative cellular automaton model for biofilms. J. Environ. Eng..

[165] Pizarro G.E., Teixeira J.o., Sepúlveda M., Noguera D.R. (2005). Bitwise implementation of a two-dimensional cellular automata biofilm model. J. Comput. Civ. Eng..

[166] Guibaud G., Tixier N., Bouju A., Baudu M. (2003). Relation between extracellular polymers’ composition and its ability to complex cd, Cu and Pb. Chemosphere.

[167] Jolley J.G., Geesey G.G., Hankins M.R., Wright R.B., Wichlacz P.L. (1989). In situ, real-time ft-ir/cir/atr study of the biocorrosion of copper by gum arabic, alginic acid, bacterial culture supernatant and *pseudomonas atlantica* exopolymer. Appl. Spectrosc..

[168] Fischer W.R., Wagner D., Siedlarek H. (1995). Microbiologically influenced corrosion in potable water installations: An engineering approach to developing countermeasures. Mater. Perform..

[169] Picioreanu C., van Loosdrecht M.C.M. (2002). A mathematical model for initiation of microbiologically influenced corrosion by differential aeration. J. Electrochem. Soc..

[170] Efird K.D. (1977). Effect of fluid dynamics on the corrosion of copper-base alloys in sea water. Corrosion.

[171] Yabuki A., Murakami M. (2008). Breakaway properties of film formed on copper and copper alloys in erosion-corrosion by mass transfer equation. Mater. Corros..

[172] Lehtola M.J., Laxander M., Miettinen I.T., Hirvonen A., Vartiainen T., Martikainen P.J. (2006). The effects of changing water flow velocity on the formation of biofilms and water quality in pilot distribution system consisting of copper or polyethylene pipes. Water Res..

[173] Malki B., Baroux B. (2005). Computer simulation of the corrosion pit growth. Corros. Sci..

[174] Ha H., Taxen C., Cong H.B., Scully J.R. (2011). Effect of applied potential on pit propagation in copper as function of water chemistry. J. Electrochem. Soc..

[175] Newman J.S. (1973). Electrochemical Systems.

[176] Seyeux A., Maurice V., Marcus P. (2013). Oxide film growth kinetics on metals and alloys I. Physical model. J. Electrochem. Soc..

